# The study on the live streaming frequency strategy choices of streamers in live E-commerce

**DOI:** 10.1371/journal.pone.0324783

**Published:** 2025-07-09

**Authors:** Yingjing Wu, Ying Cui

**Affiliations:** 1 Suqian University, suqian, China; 2 Shanghai Maritime University, Shanghai, China; Rikkyo University: Rikkyo Daigaku, JAPAN

## Abstract

Live-streaming e-commerce, emerging as a novel business model, has garnered considerable attention from leading platforms and brands. It not only exerts a profound influence on consumers’ lifestyles and consumption habits but also presents brand owners with a fresh sales model. This paper focuses on the perspective of the live-streamer and explores the scenario where a live-streamer sells two competing products through live shows. By constructing three models—single live show, two live shows with information disclosure, and two live shows without information disclosure—it is discovered that, when the proportion of fans for the live-streamer is relatively low, the live-streamer opts for the two live shows strategy without information disclosure. When fan percentage is elevated and product competition diminishes, the streamer consistently favors the single live-show strategy. As the proportion of fans is moderate and product competition diminishes, the streamer increasingly favors a two live show strategy that includes disclosed product information. Furthermore, when the live show interval is short and the proportion of live-streamer fans is small, the live-streamer prefers to adopt the two live show strategies without product information disclosure; when the live show interval is long, the live-streamer prefers the two live show strategies under product information disclosure. When there is a high proportion of fans, regardless of the length of the interval between the two live shows, the live-streamer prefers the single live show strategy.

## 1. Introduction

E-commerce live-streaming, a novel selling format recently, has been garnering more and more attention as it can effectively boost product sales. In 2023, the sales of live-streaming e-commerce accounts for almost 1/3 of total online shopping sales in China [1]. Differing from traditional e-commerce selling format without purchase time limitation, an e-commerce live-streaming session held by a streamer promoting certain products often only last for a limited duration. Given this, live-streamers may hold multiple live-streaming sessions to promote products from contracting with suppliers. Product information in live-streaming may be disclosed by the live-streamer before the live-streaming activity starts to attract more potential audiences into the live show and thus, prompt the probability of successful transaction. Specifically, live-streamer who accept multiple product promotions can choose to promote multiple products in one live show or hold multiple live shows and promote products separately and sequentially.

In the case of multiple live show, live-streamer may disclose the live-streaming product information (e.g., product selling price) before his or her first live show begins. Additionally, the live-streamer can disclose all the live shows’ product information at the beginning of the first live show, or choose to disclose the second live show product information until the finish of the first live show. Hence, this raises questions: When should the live-streamer hold multiple live shows of competitive products with brand differences? If he holds, how does the live-streamer arrange the live shows product information disclosure strategy: disclosing all at once or disclosing sequentially?

To explore the above questions, we establish a two-stage game-theoretic model consisting of a well-known band manufacturer, a small brand manufacturer, and one live-streamer. We build three different scenarios: products are promoted in a single live show (S), products are separately promoted in two live shows sequentially (the first live show promotes the well-known brand product and the second live show promotes the unfamous brand product) and the product information of the two live shows is disclosed before the beginning of the first live-show (DC-FS), and products are separately promoted in two live shows sequentially and the live show product information is disclosed sequentially (DN-FS). Regarding whether the second live show information is disclosed before the first live show begins, we also refer the DC-FS strategy as two live shows with information disclosure and the DN-FS strategy as two live shows without information disclosure.

Our results find that when the proportion of fans of the live-streamer is low, the live-streamer prefers the two live shows strategy without product information disclosure. When the proportion of fans of the live-streamer is high, with the decrease of product competition, the live-streamer is always more inclined to the single live show strategy. When the proportion of live-streamer fans is medium, with the decrease of product competition, the live-streamer is more inclined to the two live shows strategy with product information disclosure.

In the case of a short live show interval, it is always the most favorable for the live-streamer to adopt a two live show strategy, compared to the benchmark single live show model. With the increase of product competition, the live-streamer is more inclined to the two live shows strategy without product information disclosure. In the case of a relatively small proportion of live-streamer fans, when the live show interval is short, the live-streamer prefers to adopt the two live shows strategy without product information disclosure; when the live show interval is large, the live-streamer prefers the two live shows strategy under product information disclosure. In the case of a high proportion of fans, regardless of the length of the interval between the two live shows, the live-streamer prefers a single live show strategy.

## 2. Related literature

### 2.1. Live-streaming e-commerce

Our paper is studied under the context of live-streaming e-commerce and thereby is closely related to the literature on live-streaming e-commerce. Since the beginning of live e-commerce in 2016, some scholars have discussed the characteristics of live e-commerce and the motivation of consumers’ participation. Cai et al. [2] used live shows embedded in e-commerce and integrated live e-commerce to carry out live shows. Consumers have utilitarian intentions to watch live shows and hedonic intentions to watch live shows due to live-streamers in live e-commerce. Wongkitrungrueng and Assarut [3] found that the practical value and hedonic value perceived by consumers from live streaming e-commerce affect consumers’ trust in live-streamers and products, thus affecting consumers’ participation in live streaming e-commerce. Su [4] consumers mainly experience live e-commerce through ‘immersive’ experience. Li et al. [5] found that synchronization and interactivity in live e-commerce can effectively enhance user stickiness and promote the development of live e-commerce. Some scholars have focused on the study of different factors affecting consumers’ purchase intention in live e-commerce. Sun et al. [6] investigated consumers who shopped on different live e-commerce platforms through Taobao, Jingdong and Mushroom Street, and found that visibility, interactivity and shopping guidance in live e-commerce were positively correlated with consumers’ purchase intention. Zhang et al. [7] found that the quality of information and interaction in live e-commerce will affect consumers’ purchase intention in live e-commerce. Ko and Chen [8] took Facebook as the research object. The research shows that the professionalism of the live-streamer, the similarity and familiarity between consumers and the live-streamer will affect the interaction between the live show room, and then affect the consumer ‘s purchase intention. Park and Lin [9]’s live content and product fit and the live-streamer’s own fit with the product will increase consumers’ willingness to buy. The research on related issues of live e-commerce is discussed by constructing a mathematical model. Hou et al. [10] divided the consumers in the live show into naive consumers and mature consumers, and combined the main characteristics of the live-streamer (including social influence, live-streamer reliability, bargaining power) and different types of consumers’ perception of product quality to study the company’s best live show adoption strategy. Qi et al. [11] studied the capacity investment strategy of manufacturers selling products on live shopping platforms, and proposed that Pareto improvement between manufacturers and live-streamers can be achieved through commission profit sharing contracts.

However, the above literature ignores the strategy choice regarding live show sessions in promoting certain number of products and the information disclosure problem in the face of two live show activities. This paper investigates such interactions between a live-streamer and two brand product sellers/manufacturers.

### 2.2. Product brand competition

Our research contributes to the stream of literature on product brand competition. Baltas [12] studied the product choice problem of consumers under the multi-brand strategy of enterprises by analyzing consumer purchasing behavior. Some scholars focus on the impact of brand differentiation strategy on supply chain members. Luo et al. [13] investigated the impact of competition generated by brand differences on the supply chain. The study shows that similar brands are unfavorable to manufacturers and favorable to retailers. Moorthy et al. [14] investigated manufacturers’ channel selection strategies under brand differentiation in the presence of consumers’ brand preferences. Some scholars have also explored the competition between retailers’ private labels and manufacturers’ brands, and analyzed the impacts of brand competition on retailers and manufacturers. Kuo and Yang [15] considered the competition between retailers’ private labels and manufacturers’ brands, and explored the impacts of two products with brand differences on manufacturers and retailers. On this basis, Ru et al. [16] analyzed the impact of retailers’ private labels on manufacturers’ brands, and Cui et al. [17] investigated the market entry strategies of risk-averse retailers’ private labels. Nonetheless, these papers do not consider the live-streaming e-commerce selling model, which appears as a promising business ecosystem recently, and this paper explores the two sellers’ brand product competition in the context of live-streaming e-commerce.

### 2.3. Information disclosure

Our study also connects to the stream of research on information disclosure. Ghosh et al. [18] investigated the effects of consumer attention and search cost on firms’ qualitative information disclosure. It was found that companies tend to disclose less information when consumer attention is high or search cost is high. Chenavaz et al. [19] found after research that when a company’s product quality is high, the company should choose to disclose more information about product quality. Guan et al. [20] considered the impact of disclosure cost on the disclosure strategies of manufacturers and retailers by taking product quality as an endogenous variable. Johnson and Myatt [21] find that firms can make higher profits when they display product information completely or not at all, and Boleslavsk and Said [22] consider the impact of the degree of information disclosure on consumer demand and the optimal pricing decisions of firms for a new product in a competitive situation. Zhang [23] analyzed the impact of the degree of information disclosure on consumer valuation in the presence of competition, as well as the disclosure strategies of different products. Our paper complements the above studies by studying a live-streamer’s product information disclosure problem in a two live shows selling setting.

In the market environment where live show e-commerce is developing rapidly, how to choose the optimal product live show strategy has become a problem that live-streamers need to pay attention to. In order to study the optimal decision-making problem of live-streamers in the context of live show e-commerce, based on the current situation of live show e-commerce development, combined with reality, from the perspective of live-streamers, considering the proportion of live-streamer fans, the degree of product competition and the interval of live show, this paper studies and analyzes the selection of live show strategy and product live show order strategy of live-streamers in different situations, and seeks the decision-making choice that is most conducive to live-streamers. It provides certain reference value and management reference for the future decision-making of how to arrange the live show strategy of the product.

## 3. Problem description and model assumptions

It is assumed that the market consists of two brand product sellers/manufacturers providing competitive products and one live-streamer. In the rest of the paper, we use the term “seller” and “manufacturer” interchangeably. We assume that one brand seller is large-size and famous, and the other brand seller is small and unfamous, with both sellers selling the same type of products. The well-known brand seller (“seller 1”) provides product a to the live-streamer for live sales at the price $p_{a}$. The niche brand seller (“seller 2”) provides the product b to the live-streamer at the price $p_{b}$. At the same time, brand seller 1 and brand seller 2 need to pay the live show a fixed fee to the live-streamer, respectively, that is, the fixed “pit fee” $F_{1}$ and $F_{2}$. Furthermore, they also need to pay the commission fee with the commission ratio of $\gamma_{1}$ and $\gamma_{2}$, respectively. We assume that the pit fee $F_{i}$ and commission rate $\gamma_{i}$ are exogenously given because they are determined before the live-streamer and sellers’ decisions we consider in this paper, that is, band sellers’ selling price and live-streamer’s sales effort.

In reality, the live-streamer charges the same pit fees and commissions rate for the same type of products. Since this paper studies two products of the same type, the live-streamer charges the same pit fee and commission rate for the two products. Therefore, we assume that ${{F=F}_{1}=F}_{2}$, ${\gamma =\gamma }_{1}=\gamma_{2}$. It is assumed that the live-streamer sells two products through the live show(s). For the live-streamer, the live-streamer can choose to sell product a and product b simultaneously in a single live show, or choose to respectively sell product a and product b in two sequential live shows, that is, the well-known brand product a is sold in the first live show, and the niche brand product b is sold in the second live show. We refer to such choice of the live-streamer as “live show session strategy”. Turning the product order in the two live shows does not qualitatively change our result and fixing the product display sequence can make us focus on the live-streamer’s live show session decision and information disclosure decision. Combined with reality, when the live-streamer puts the two products in two live shows, the live-streamer can disclose the product information of the two live shows in advance. In this case, consumers will know the product information of the two live shows at the same time. We refer to this case as “two live shows with information disclosure”. The live-streamer can also disclose the product information of the second live show after the end of the first live show, that is, the product information is disclosed at different times. At this time, consumers cannot know the product information of the two live shows at the same time. We refer to this situation as “two live shows without information disclosure”.

**Fig 1:**
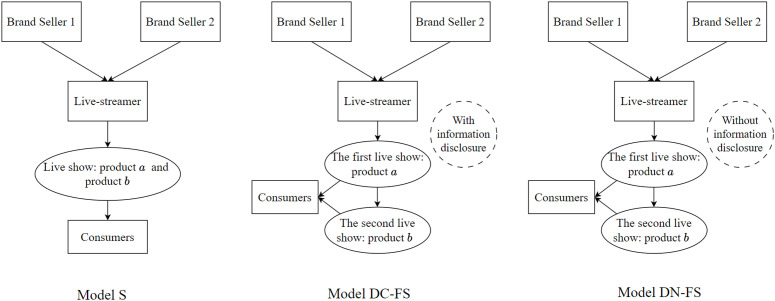
Model structure (Prepared by the authors).

Based on the above different situations, we construct three models, As shown in Fig 1: (1) single live show (Model S); (2) Two live shows under product information disclosure (model DC-FS); (3) Two live shows without product information disclosure (model DN-FS). Among them, DC represents the model under product information disclosure, that is, to publish the product information of the two live shows to consumers at the same time, DN represents the model under product information undisclosed, that is, to publish the product information of the two live show rooms to consumers at different times; F means well-known brand products, S means niche brand products, and FS means that the live-streamer puts the well-known brand products on the first live show and the niche brand products on the second live show.

Suppose that the number of potential consumers in the live show is N. Considering the influence of the number of fans on the sales of live show products, all potential consumers are divided into two types: fan consumers and non-fan consumers. Fan-type consumers have great trust in the live-streamer and its recommended products, and will purchase all products that make their utility positive in the live show; consumers who are not fans are relatively rational in live e-commerce, and such consumers will buy the products that make them most effective in the live show. Assuming that the proportion of fan-type consumers is φ(0<φ<1), and thus the number of fan-type consumers in the live show room is φN; the proportion of non-fan-type consumers is 1−φ, and thereby the number of non-fan-type consumers in the live show room is (1−φ)N. In order to simplify the model, let the number of potential consumers N=1. Consumers have stronger brand trust in well-known brand products and weaker brand trust in niche brand products. It is assumed that the consumer’s valuation of the well-known brand product a is V with V follows uniform distribution on [0,1], i.e., V~U[0,1], and the valuation of the niche brand product b is θV, where θ(0<θ<1) is the brand discount level of the consumer to the niche brand product b, which can also indicate the degree of competition between the two products. In live e-commerce, fan-type consumers, because they are fans of the live-streamer, trust the live-streamer and their recommended products more, so the valuation of products will be higher than the real value level of products because they like and trust the live-streamer. It is assumed that fan consumers have a spillover level α(α>1) for the valuation of live show products [24]. We assume that α is fixed since the live-streamer’s ability in promoting products is relatively stable and will not significant change during the live shows. Compared with fan-type consumers, non-fan-type consumers are more rational when purchasing products in the live show. Their valuation of the product is the true value of the product. Therefore, the valuation of fan consumers on well-known brand product a is αV, and the valuation of niche brand product b is αθV; non-fan consumers’ valuation of well-known brand product a is V, and the valuation of niche brand product b is θV.

When consumers buy products in the live show, in addition to the utility of the product itself, in the live show process, the interactivity and entertainment experienced by consumers in the live show due to the efforts of the live-streamer will also bring certain additional utility to consumers [24]. Taking into account the different purchasing behaviors between fan consumers and non-fan consumers, when fan consumers purchase products in the live show, they will pay attention to the additional utility brought by the live-streamer in addition to the utility of the product itself, while non-fan consumers pay more attention to the utility of the product itself. Therefore, the extra utility of the live-streamer’s efforts to fan-type consumers is greater, and the extra utility to non-fan-type consumers is smaller. For fan-type consumers, It is assumed that the additional utility brought by the live-streamer to the consumers due to the effort during the live show is $e_{i}(i=a,b)$, which equals the entertainment effort exerted by the live-streamer, and the additional utility brought by the live-streamer for different products in the same live show (i.e., in scenario S) is the same. Note that $e_{i}$ represents the additional utility from the live-streamer’s entertainment effort to boost sales such as singing, dancing, or telling stories in the live show. For non-fan-type consumers, in order to simplify the model, it is assumed that the extra utility of the live-streamer to non-fan consumers is 0. Assuming that the non-fan-type consumers’ extra utility due to sales effort is larger than zero will not qualitatively affect our main result but will complicate our analysis and make the model intractable. Furthermore, since non-fan-type consumers are relatively reasonable to product purchase decision, live-streamer’s entertainment sales effort such as singing, telling story, or dancing may not influence these consumers’ purchase decision, justifying the assumption above. The effort cost of the live-streamer in the live show is $C=\frac{1}{2}ke_{i}^{2}(i=a,b)$ [1], where k(k>0) is the effort cost coefficient.

When the live-streamer conducts two live shows to sell the two products, fan consumers are willing to wait and watch each live show of the live-streamer due to their trust and preference for the live-streamer. Therefore, the live-streamer’s placing the product on a single live show or two live shows has no effect on fan-type consumers. For non-fan consumers, if non-fan consumers fail to buy products in the first live show, they need to wait and enter the next live show. On the contrary, if non-fan consumers buy products in the first live show, they do not need to wait to enter the next live show. At this time, non-fan consumers who bought products in the first live show will have a certain positive effect because they do not need to wait for the next live show. It is assumed that the positive utility brought to non-fan consumers by waiting for cost savings is s(s>0). The parameter s also reflects the time interval between the first live show and the second live show, as a higher s means a larger time interval between the two live shows. Considering the difference between live-streaming e-commerce and other shopping channels, the motivation of consumers to watch and use live-streaming e-commerce includes not only pragmatism with the intention of purchasing products, but also hedonism with the intention of fun and entertainment obtained by watching live shows. For consumers, the utility of watching live show is greater than the time and cost of watching live show. Therefore, it is assumed that all consumers will enter the live show and watch the live show. The hypothesis in this paper is based on the actual situation.

In the case of adopting the two live shows strategy, after the first live show, some non-fan-type consumers will be transformed into fan-type consumers due to their preference for the live show style of the live-streamer or their recommended products. The number of fans of the live-streamer will increase to varying degrees after each live show. To simplify the model, this paper only considers the transformation of non-fan consumers into fan consumers, without considering the transformation of fan consumers into non-fan consumers. It is assumed that non-fan consumers will be transformed into fan consumers with a probability of ρ(ρ>0) after watching the first live show of the live-streamer. We also assume that ρ is exogenous given owing to the relatively stable entertainment ability of the live-streamer in attracting consumers.

The symbols and definitions involved in this article are shown in Table 1.

**Table 1:** Symbols and meanings.

Symbolic	Meaning
V	Consumers’ valuation of product a, V~U[0,1]
N	Number of Potential Consumers in live shows
φ	The proportion of fans consumers, 0<φ<1
α	The spillover level of fan consumers on product valuation, α>1
θ	Consumers’ brand discount level for niche brand product b,0<θ<1
s	The positive effect of non-fan consumers waiting for savings, s>0
ρ	The probability of non-fan consumers turning into fans, 0<ρ<1
k	Live-streamer effort cost coefficient, k>0
γ	Commission rate, 0<γ<1
F	Pit fee
$p_{i}(i=a,b)$	The price of product i, decision variables
$e_{i}(i=a,b)$	The additional utility and decision variables that the live-streamer brings to fan consumers when selling product i live.
$D_{F_{i}}^{S,DC,DN}(i=a,b)$	Fan consumers’ demand for product i under different models
$D_{N_{i}}^{S,DC,DN}(i=a,b)$	Non-fan consumers’ demand for product i under different models
$\pi_{L}^{S,DC,DN}$	Live-streamer’s profit under different models
$\pi_{1,2}^{S,DC,DN}$	The profits of brand seller 1 and brand seller 2 under different models

## 4. Model Analysis

### 4.1. Single live show (Model S)

In the single live show scenario (Model S), the live-streamer sells the well-known brand product a and the niche brand product b in a single live show. Because the extra utility brought by the live-streamer for different products to the fan-type consumers in the same live show is the same. Therefore, it is assumed that the additional utility brought by the live-streamer to the fan consumers in the single live show is $e_{a}=e_{b}=e$. In Model S, the live-streamer sells the product in a single live show, and non-fan consumers do not need to wait for the next live show. This positive effect on non-fan consumers due to waiting for cost savings is s(s>0). Subscript F is used to represent fan consumers, and subscript N is used to represent non-fan consumers. In model S, the utility of fan-type consumers purchasing product a and product b in the live show is respectively as follows:


$$U_{F_{a}}^{S}=\alpha V-p_{a}+e;$$ (4−1)

$$U_{F_{b}}^{S}=\alpha \theta V-p_{b}+e.$$ (4-2)

The utility obtained by non-fan consumers to purchase product a and product b in the live show is respectively as follows:


$$U_{N_{a}}^{S}=V-p_{a}+s;$$ (4-3)

$$U_{N_{b}}^{S}=\theta V-p_{b}+s.$$ (4-4)

Fan-type consumers are fans of the live-streamer and have great trust in the live-streamer and its recommended products, so fan-type consumers will purchase all products that make their utility positive in the live show room. That is, fan-type consumers may buy both product a and product b. When $U_{F_{a}}^{S}\geq 0$, fan consumers will buy well-known brand product a; when $U_{F_{b}}^{S}\geq 0$, fans will buy niche brand product b. Compared with fan-type consumers, non-fan-type consumers are more rational when purchasing products in the live show room. Such consumers will compare the utility of the two products and select the most effective product to buy. When $U_{N_{a}}^{S}\geq U_{N_{b}}^{S}$ and $U_{N_{a}}^{S}\geq 0$, non-fan consumers will buy well-known brand product a; when $U_{N_{b}}^{S}\geq U_{N_{a}}^{S}$ and $U_{N_{b}}^{S}\geq 0$, non-fan consumers will buy niche brand product b.

Therefore, in model S, the demands of fan-type consumers for well-known brand product a and niche brand product b are:


$$D_{F_{a}}^{S}=\left(1-\frac{p_{a}-e}{\alpha }\right)\varphi ;$$ (4-5)

$$D_{F_{b}}^{S}=\left(1-\frac{p_{b}-e}{\alpha \theta }\right)\varphi .$$ (4-6)

The needs of non-fan consumers for well-known brand product a and niche brand product b are:


$$D_{N_{a}}^{S}=\left(1-\frac{p_{a}-p_{b}}{1-\theta }\right)(1-\varphi );$$ (4-7)

$$D_{N_{b}}^{S}=\left(\frac{p_{a}-p_{b}}{1-\theta }-\frac{p_{b}-s}{\theta }\right)(1-\varphi ).$$ (4-8)

In Model S, the total demand of well-known brand product a is:


$$D_{a}^{S}=D_{F_{a}}^{S}+D_{N_{a}}^{S}=\left(1-\frac{p_{a}-e}{\alpha }\right)\varphi +\left(1-\frac{p_{a}-p_{b}}{1-\theta }\right)(1-\varphi ).$$ (4-9)

The total demand of niche brand product b is:


$$D_{b}^{S}=D_{F_{b}}^{S}+D_{N_{b}}^{S}=\left(1-\frac{p_{b}-e}{\alpha \theta }\right)\varphi +\left(\frac{p_{a}-p_{b}}{1-\theta }-\frac{p_{b}-s}{\theta }\right)(1-\varphi ).$$ (4-10)

In Model S, the profit of the live-streamer is:


$$\pi_{L}^{S}=\gamma p_{a}D_{a}^{S}+F-\frac{1}{2}ke^{2}+\gamma p_{b}D_{b}^{S}+F-\frac{1}{2}ke^{2}.$$ (4-11)

The profits of brand seller 1 and seller 2 are as follows:


$$\pi_{1}^{S}=(1-\gamma )p_{a}D_{a}^{S}-F;$$ (4-12)

$$\pi_{2}^{S}=(1-\gamma )p_{b}D_{b}^{S}-F.$$ (4-13)

Among them,


$$D_{a}^{S}=\left(1-\frac{p_{a}-e}{\alpha }\right)\varphi +\left(1-\frac{p_{a}-p_{b}}{1-\theta }\right)(1-\varphi );$$

$$D_{b}^{S}=\left(1-\frac{p_{b}-e}{\alpha \theta }\right)\varphi +\left(\frac{p_{a}-p_{b}}{1-\theta }-\frac{p_{b}-s}{\theta }\right)(1-\varphi ).$$

Using the backward induction solution method, we can get the optimal solution of brand seller 1, 2 and live-streamer under the model S as follows:


$$p_{a}^{S}=\frac{-2k\alpha^{2}(-1+\theta )(\gamma (-1+\theta )\varphi^{2}(-2+s{\left(-1+\varphi \right)}^{2}+\theta \varphi )+2k\alpha \theta (2(-1+\theta )\varphi +\alpha (-1+\varphi )(2+s{\left(-1+\varphi \right)}^{2}+(-2+\theta )\varphi )))}{A_{1}(k,\gamma ,\alpha ,\theta ,\varphi )};$$

$$p_{b}^{S}=\frac{\begin{array}{c} 2k\alpha^{2}(-1+\theta )\theta (-\gamma (-1+\theta )\varphi^{2}(-1+2s{\left(-1+\varphi \right)}^{2}+2\theta \varphi )\\ +2k\alpha (-\alpha \theta +\theta (-2+\alpha +2\theta )\varphi^{2}+2s{\left(-1+\varphi \right)}^{2}(-\alpha +(-1+\alpha +\theta )\varphi ))) \end{array}}{A_{2}(k,\gamma ,\alpha ,\theta ,\varphi )};$$

$$e^{S}=\frac{\alpha \gamma (-1+\theta )\varphi (-\gamma (-1+\theta )\varphi^{2}(1+s{\left(-1+\varphi \right)}^{2}+\theta \varphi )+A_{1}^{S}(k,\gamma ,\alpha ,\theta ,\varphi ,s))}{A_{2}(k,\gamma ,\alpha ,\theta ,\varphi )}.$$

$A_{1}(k,\gamma ,\alpha ,\theta ,\varphi )$, $A_{2}(k,\gamma ,\alpha ,\theta ,\varphi )$, and $A_{1}^{S}(k,\gamma ,\alpha ,\theta ,\varphi ,s)$ are shown in the S1 Appendix.

### 4.2. Two live shows under product information disclosure (Model DC-FS)

In the model DC-FS, the live-streamer puts the products in two live shows, in which the well-known brand product a is sold in the first live show, the niche brand product b is sold in the second live show, and the product information of the two live shows is disclosed at the same time. At this time, consumers can know the product information of the two live shows at the same time before the first live show starts. In the two live shows, fan consumers will enter the two live shows of the live-streamer because of their trust and love for the live-streamer, and purchase products that make their utility positive. Since consumers know the product information of the two live shows at the same time, non-fan consumers will still compare the utility of the two live shows and purchase the product that maximizes its utility. The additional utility brought by the live-streamer to the fan consumers in the two live shows is$e_{a}ande_{b}$, respectively. The positive utility of non-fan consumers buying product a in the first live show without waiting for the second live show is s.

In the model DC-FS, the utilities of two types of consumers buying well-known brand product a in the first live show are:


$$U_{F_{a}}^{DC-FS}=\alpha V-p_{a}+e_{a};$$ (4-14)

$$U_{N_{a}}^{DC-FS}=V-p_{a}+s.$$ (4-15)

The utilities obtained by the two types of consumers in purchasing niche brand product b in the second live show are:


$$U_{F_{b}}^{DC-FS}=\alpha \theta V-p_{b}+e_{b};$$ (4-16)

$$U_{N_{b}}^{DC-FS}=\theta V-p_{b}.$$ (4-17)

When $U_{F_{a}}^{DC-FS}\geq 0$, fan-type consumers will buy well-known brand products a in the first live show; when $U_{N_{a}}^{DC-FS}\geq U_{N_{b}}^{DC-FS}$ and $U_{N_{a}}^{DC-FS}\geq 0$, non-fan consumers will buy well-known brand producta in the first live show.

Therefore, in the first live show, the two types of consumers’ demand for well-known brand product a are:


$$D_{F_{a}}^{DC-FS}=\left(1-\frac{p_{a}-e_{a}}{\alpha }\right)\varphi ;$$ (4-18)

$$D_{N_{a}}^{DC-FS}=\left(1-\frac{p_{a}-p_{b}-s}{1-\theta }\right)(1-\varphi ).$$ (4-19)

When $U_{F_{b}}^{DC-FS}\geq 0$, fan-type consumers will buy niche brand products bin the second live show; when $U_{N_{b}}^{DC-FS}\geq U_{N_{a}}^{DC-FS}$ and $U_{N_{b}}^{DC-FS}\geq 0$, non-fan consumers will wait and enter the second live show to buy niche brand product b. In reality, after each live show, non-fan-type consumers will be transformed into fan-type consumers due to their preference for the live show style of the live-streamer or their recommended products. The number of fans of the live-streamer will increase to varying degrees after each live show. In order to simplify the model, this paper only considers the transformation of non-fan consumers into fan consumers, without considering the transformation of fan consumers into non-fan consumers. It is assumed that non-fan consumers will be transformed into fan consumers with a probability of ρ(ρ>0) after watching the first live show of the live-streamer. After the first live show, it can be subdivided into three categories according to the purchase behavior of non-fan consumers: Purchase well-known brand products ain the first live show; buy niche brand products b in the second live show; neither product is purchased. These three types of non-fan consumers have a certain probability of turning into fan consumers after watching the first live show. It is assumed that the conversion probabilities of these three types of non-fan consumers who are converted into fans after the first live show and non-fan consumers who intend to buy niche brand products b in the second live show and do not convert into fans will enter the second live show. Therefore, according to the hypothesis, the number of fan consumption in the second live show is [ϕ+ρ(1−φ)].The number of non-fan consumers who still maintain the purchase behavior of non-fan consumers in the second live show is (1−ρ)(1−φ).

Therefore, in the second live show, the two types of consumers’ demand for niche brand product b are:


$$D_{F_{b}}^{DC-FS}=\left(1-\frac{p_{b}-e_{b}}{\alpha \theta }\right)\left[\phi +\rho (1-\varphi )\right];$$ (4-20)

$$D_{N_{b}}^{DC-FS}=\left(\frac{p_{a}-p_{b}-s}{1-\theta }-\frac{p_{b}}{\theta }\right)(1-\rho )(1-\varphi ).$$ (4-21)

In the model DC-FS, the total demand of the well-known brand product a in the first live show is:


$$D_{a}^{DC-FS}=\left(1-\frac{p_{a}-e_{a}}{\alpha }\right)\varphi +\left(1-\frac{p_{a}-p_{b}-s}{1-\theta }\right)(1-\varphi ).$$ (4-22)

The total demand of minority brand product b in the second live show is as follows:


$$D_{b}^{DC-FS}=\left(1-\frac{p_{b}-e_{b}}{\alpha \theta }\right)\left[\varphi +\rho (1-\varphi )\right]+\left(\frac{p_{a}-p_{b}-s}{1-\theta }-\frac{p_{b}}{\theta }\right)(1-\rho )(1-\varphi ).$$ (4-23)

In the model DC-FS, the live-streamer sells the well-known brand product a in the first live show and the niche brand product b in the second live show, and discloses the product information of the two live shows at the same time. First, brand seller 1 and brand seller 2 simultaneously decide the price $p_{a}$ and $p_{b}$ of products a and b, and then the live-streamer decides its effort level $e_{a}$ in the first live show, and decides its effort level $e_{b}$ in the second live show. In the model DC-FS, the profit of the live-streamer is:


$$\pi_{L}^{DC-FS}=\gamma p_{a}D_{a}^{DC-FS}+F-\frac{1}{2}ke_{a}^{2}+\gamma p_{b}D_{b}^{DC-FS}+F-\frac{1}{2}ke_{b}^{2}.$$ (4-24)

The profits of brand sellers 1 and 2 are as follows:


$$\pi_{1}^{DC-FS}=(1-\gamma )p_{a}D_{a}^{DC-FS}-F;$$ (4-25)

$$\pi_{2}^{DC-FS}=(1-\gamma )p_{b}D_{b}^{DC-FS}-F.$$ (4-26)

Among them,


$$D_{a}^{DC-FS}=\left(1-\frac{p_{a}-e_{a}}{\alpha }\right)\varphi +\left(1-\frac{p_{a}-p_{b}-s}{1-\theta }\right)(1-\varphi ),$$

$$D_{b}^{DC-FS}=\left(1-\frac{p_{b}-e_{b}}{\alpha \theta }\right)\left[\varphi +\rho (1-\varphi )\right]+\left(\frac{p_{a}-p_{b}-s}{1-\theta }-\frac{p_{b}}{\theta }\right)(1-\rho )(1-\varphi ).$$

Using the reverse order solution method, we can get the optimal solution of brand 1, 2 and live-streamer under the model DC-FS as follows:


$$p_{a}^{DC-FS}=\frac{\left(1-\gamma )(1-\varphi )(s-\rho -s\rho +\theta \rho +(1+s-\theta )(-1+\rho )\varphi )-A_{1}^{DC-FS}(k,\gamma ,\alpha ,\theta ,\varphi ,s,\rho \right)}{A_{3}(k,\gamma ,\alpha ,\theta ,\varphi ,\rho )},$$

$$p_{b}^{DC-FS}=\frac{k\alpha^{2}\theta^{2}A_{2}^{DC-FS}(k,\gamma ,\alpha ,\theta ,\varphi ,s,\rho )}{A_{4}(k,\gamma ,\alpha ,\theta ,\varphi ,\rho )},$$

$$e_{a}^{DC-FS}=\frac{\gamma \varphi ((1-\gamma )(1-\varphi )(s-\rho -s\rho +\theta \rho +(1+s-\theta )(-1+\rho )\varphi )-A_{1}^{DC-FS}(k,\gamma ,\alpha ,\theta ,\varphi ,s,\rho ))}{k\alpha A_{3}(k,\gamma ,\alpha ,\theta ,\varphi ,\rho )},$$

$$e_{b}^{DC-FS}=\frac{\alpha \gamma \theta (\rho +\varphi -\rho \varphi )A_{2}^{DC-FS}(k,\gamma ,\alpha ,\theta ,\varphi ,s,\rho )}{A_{4}(k,\gamma ,\alpha ,\theta ,\varphi ,\rho )}.$$

$A_{3}(k,\gamma ,\alpha ,\theta ,\varphi ,\rho )$, $A_{4}(k,\gamma ,\alpha ,\theta ,\varphi ,\rho )$, and $A_{1}^{DC-FS}(k,\gamma ,\alpha ,\theta ,\varphi ,s,\rho )$ are shown in the S1 Appendix.

### 4.3. Two live shows without product information disclosure (Model DN-FS)

In the model DN-FS, the live-streamer still puts the products in two live shows, sells the well-known brand product ain the first live show, and sells the niche brand product b in the second live show, but the live-streamer does not disclose the product information of the two live shows at the same time. At this time, consumers cannot know the product information of the two live shows at the same time. It is also assumed that all consumers will enter and watch the first live show. In the model DN-FS, fan consumers will still enter the two live shows of the live-streamer and buy products that make their utility positive. Non-fan consumers cannot make purchases by comparing the utility of the two products because they cannot know the product information of the two live shows at the same time. Therefore, in the model DN-FS, non-fan consumers will also buy products that make their utility positive. Different from fan consumers, non-fan consumers will not buy two kinds of products of the same type, that is, if non-fan consumers buy well-known brand product a in the first live show, in the second live show, if non-fan consumers who bought well-known brand product a in the first live show do not convert into fan consumers, then such non-fan consumers will not enter the second live show to buy niche brand product b.

Consistent with the model DC-FS, in the model DN-FS, the utilities obtained by the two types of consumers to purchase the well-known brand product a in the first live show are:


$$U_{F_{a}}^{DN-FS}=\alpha V-p_{a}+e_{a};$$ (4-27)

$$U_{N_{a}}^{DN-FS}=V-p_{a}+s.$$ (4-28)

The utilities obtained by the two types of consumers in purchasing niche brand product b in the second live show are:


$$U_{F_{b}}^{DN-FS}=\alpha \theta V-p_{b}+e_{b};$$ (4-29)

$$U_{N_{b}}^{DN-FS}=\theta V-p_{b}.$$ (4-30)

When $U_{F_{a}}^{DN-FS}\geq 0$, fan-type consumers will buy a well-known brand a in the first live show; when $U_{N_{a}}^{DN-FS}\geq 0$, non-fan consumers will buy well-known brand product a in the first live show.

Therefore, in the first live show, the two types of consumers’ demand for well-known brand product a are:


$$D_{F_{a}}^{DN-FS}=\left(1-\frac{p_{a}-e_{a}}{\alpha }\right)\varphi ,$$ (4-31)

$$D_{N_{a}}^{DN-FS}=\left[1-\left(p_{a}-s\right)\right](1-\varphi ).$$ (4-32)

When $U_{F_{b}}^{DN-FS}\geq 0$, fan-type consumers will buy a well-known brand bin the second live show; when $U_{N_{b}}^{DN-FS}\geq 0$, non-fan consumers will buy well-known brand productb in the second live show. It is also considered that non-fan consumers will be transformed into fan consumers with the probability of ρ(ρ>0) after watching the first live show, and if non-fan consumers buy well-known brand product a in the first live show and do not transform into fan consumers, such non-fan consumers will not enter the second live show to buy niche brand product b. According to the previous hypothesis, in the model DN-FS, the number of fan consumers in the second live show is [φ+ρ(1−φ)], and the number of non-fan consumers is $\left[\left(p_{a}-s\right)-\rho \right](1-\varphi )$.

Therefore, in the second live show, the needs of the two types of consumers for niche brand product b are:


$$D_{F_{b}}^{DN-FS}=\left(1-\frac{p_{b}-e_{b}}{\alpha \theta }\right)\left[\varphi +\rho (1-\varphi )\right],$$ (4-33)

$$D_{N_{b}}^{DN-FS}=\left(1-\frac{p_{b}}{\theta }\right)\left[\left(p_{a}-s\right)-\rho \right](1-\varphi ).$$ (4-34)

In the model DN-FS, the total demand of the well-known brand product a in the first live show is:


$$D_{a}^{DN-FS}=\left(1-\frac{p_{a}-e_{a}}{\alpha }\right)\varphi +\left[1-\left(p_{a}-s\right)\right](1-\varphi ).$$ (4-35)

The total demand of minority brand product b in the second live show is as follows:


$$D_{b}^{DN-FS}=\left(1-\frac{p_{b}-e_{b}}{\alpha \theta }\right)\left[\varphi +\rho (1-\varphi )\right]+\left(1-\frac{p_{b}}{\theta }\right)\left[\left(p_{a}-s\right)-\rho \right](1-\varphi ).$$ (4-36)

In the model DN-FS, the live-streamer also sells the well-known brand product a in the first live show, and the niche brand product b is sold in the second live show, but does not disclose the product information of the two live shows at the same time. First, brand seller 1 and brand seller 2 simultaneously decide the price $p_{a}$ and $p_{b}$ of products a and b, and then the live-streamer decides its effort level $e_{a}$ in the first live show, and decides its effort level $e_{b}$ in the second live show. In the model DN-FS, the profit of the live-streamer is:


$$\pi_{L}^{DN-FS}=\gamma p_{a}D_{a}^{DN-FS}+F-\frac{1}{2}ke_{a}^{2}+\gamma p_{b}D_{b}^{DN-FS}+F-\frac{1}{2}ke_{b}^{2}.$$ (4-37)

The profits of brand sellers 1 and 2 are as follows:


$$\pi_{1}^{DN-FS}=(1-\gamma )p_{a}D_{a}^{DN-FS}-F,$$ (4-38)

$$\pi_{2}^{DN-FS}=(1-\gamma )p_{b}D_{b}^{DN-FS}-F.$$ (4-39)

Among them,


$$D_{a}^{DN-FS}=\left(1-\frac{p_{a}-e_{a}}{\alpha }\right)\varphi +\left[1-\left(p_{a}-s\right)\right](1-\varphi ),$$

$$D_{b}^{DN-FS}=\left(1-\frac{p_{b}-e_{b}}{\alpha \theta }\right)\left[\varphi +\rho (1-\varphi )\right]+\left(1-\frac{p_{b}}{\theta }\right)\left[\left(p_{a}-s\right)-\rho \right](1-\varphi ).$$

By using the backward induction solution method, the optimal solutions of brand seller 1, 2 and live-streamer under the model DN-FS can be obtained as follows:


$$p_{a}^{DN-FS}=\frac{k\alpha^{2}(-1+s(-1+\varphi ))}{2k\alpha (\alpha (-1+\varphi )-\varphi )+2\gamma \varphi^{2}},$$

$$p_{b}^{DN-FS}=\frac{k\alpha^{2}\theta^{2}A_{1}^{DN-FS}(k,\gamma ,\alpha ,\theta ,\varphi ,s)}{A_{5}(k,\gamma ,\alpha ,\theta ,\varphi ,s,\rho )},$$

$$e_{a}^{DN-FS}=\frac{\alpha \gamma (-1+s(-1+\varphi ))\varphi }{2k\alpha (\alpha (-1+\varphi )-\varphi )+2\gamma \varphi^{2}},$$

$$e_{b}^{DN-FS}=\frac{-(\alpha \gamma \theta (\rho (-1+\varphi )-\varphi )A_{1}^{DN-FS}(k,\gamma ,\alpha ,\theta ,\varphi ,s)}{A_{5}(k,\gamma ,\alpha ,\theta ,\varphi ,s,\rho )}$$

$A_{5}(k,\gamma ,\alpha ,\theta ,\varphi ,s,\rho )$ and $A_{1}^{DN-FS}(k,\gamma ,\alpha ,\theta ,\varphi ,s)$ are shown in the S1 Appendix.

**Lemma 1.** In model S, the effort level $e^{S}$of the live-streamer increases with the decrease of θ. In the model DC-FS, the effort level of the live-streamer $e_{a}^{DC-FS},e_{b}^{DC-FS}$ increases with the decrease of θ. In the model DN-FS, the effort level $e_{a}^{DN-FS}$ of the live-streamer is independent of θ, and $e_{b}^{DN-FS}$ increases with the decrease of θ.

Lemma 1 shows that under the two live show strategies (Model S, Model DC) of single live show and product information disclosure, as θ decreases, the amount of effort required by the live-streamer increases accordingly. In the single live show (Model S), the smaller the θ, the smaller the utility obtained by consumers from the niche brand product b, and the smaller the consumer demand for the niche brand product b. Because the effort level of the live-streamer will bring some additional utility to the fan-type consumers, the higher effort level of the live-streamer will make the fan-type consumers get more additional utility in the live show, thus increasing the demand of the fan-type consumers for niche brand products b. In the end, consumers have a certain demand for products with large differences between the two brands in the same live show.

In the two live shows under the product information disclosure (model DC-FS), the live-streamer puts the well-known brand product a on the first live show, the niche brand product b on the second live show, and discloses the product information at the same time. In the first live show, the live-streamer sold well-known brand products a. When θ is smaller, the price of well-known brand product a will increase. At this time, the live-streamer needs a higher level of effort to increase the effectiveness of consumers purchasing well-known brand products a in the first live show, and reduce the negative impact of reduced demand due to the increase in the price of well-known brand products, thereby increasing consumers ‘ demand for well-known brand products a. However, in the second live show, the live-streamer sold niche brand products b. When θ is smaller, the competition between products is smaller, and the utility of consumers purchasing niche brand products b in the second live show is smaller. In this case, the live-streamer needs to pay a higher level of effort to make up for the adverse effects of brand differences on product b, thereby increasing consumer demand for niche brand products b. In the two live shows without product information disclosure (model DN-FS), the live-streamer also places well-known brand products a on the first live show and niche brand products b on the second live show, but does not disclose product information at the same time. At this time, consumers cannot know the product information of the two live shows at the same time. For well-known brand products a in the first live show, consumers only know the product information of well-known brand products a in the first live show, but do not know the product information of minority brand products b in the second live show. Therefore, the well-known brand product a is equivalent to a monopoly situation. When consumers decide whether to purchase the well-known brand product a, they only need to consider the utility obtained by purchasing the well-known brand product a at present, and have nothing to do with the niche brand product b. An live-streamer does not need to consider niche brand products b when deciding the effort level of the first live show. Therefore, the effort level of the live-streamer in the first live show has nothing to do with θ.

Overall, under either the single live show scenario or the two live shows with information disclosure scenario, as the brand difference (i.e., measured by θ) enlarges (θ decreasing), more consumers are attracted by the well-known product a and thus less consumers are willing to buy product b. To reduce the negative effect caused by the diminished demand of product b, the live-streamer needs to exert more entertainment effort. Therefore, for the live-streamer, in practice, he needs to carefully introduce competing products that have a large brand difference in value because more entertainment effort is required for the him to enhance the demand of the low value one.

**Lemma 2.** (i) In model S, $\pi_{L}^{S}$ decreases with the increase of θ; (ii) In the model DC-FS, there exists $\theta^{*}$, when $\theta =\theta^{*}$, $\pi_{L}^{DC-FS}$ is the largest; (iii) In the DN-FS model, $\pi_{L}^{DN-FS}$ increases with the increase of θ.

Lemma 2 (i) shows that when the live-streamer sells two products in a single live show, the profit of the live-streamer will decrease with the increase of θ, that is, fierce product competition will damage the interests of the live-streamer. In the single live show (Model S), as θ increases, the utility obtained by consumers from niche brand products b increases. Due to the difference in purchasing behavior between fan consumers and non-fan consumers in the live show, fan consumers will increase the demand for niche brand products b, and non-fan consumers will transfer some of the demand for well-known brand products a to niche brand products b. The price of well-known brand product a and niche brand product b will decrease with the decrease of brand difference, and the effort level of live-streamer will decrease with the increase of θ. The negative impact of the reduction of consumer demand for well-known brand products a and the price reduction of well-known brand products a and niche brand products b is greater than the positive impact of the increase of niche brand products b demand and the decrease of live-streamer effort cost on live-streamer profit. Therefore, in a single live show (Model S), the profit of the live-streamer will decrease with the increase of θ, and fierce product competition will have a negative impact on the live-streamer. In reality, live-streamers often do not sell two competitive products at the same time in a single live show.

Lemma 2 (ii) shows that when the live-streamer sells the product in two live shows and discloses the product information at the same time, when $0<\theta <\theta^{*}$, the profit of the live-streamer will increase with the increase of θ. When $\theta^{*}<\theta <1$, the profit of the live-streamer will decrease with the increase of θ. In the two live shows of product information disclosure (model DC-FS), with the increase of θ, that is, the competition between niche brand products b and well-known brand products a is gradually fierce, fan consumers will increase their demand for niche brand products b, while non-fan consumers will transfer some of their demand for well-known brand products a to niche brand products b. At the same time, the live-streamer’s effort level in both live shows will decrease with the increase of θ. When $0<\theta <\theta^{*}$, that is, the degree of competition between products is relatively small, the positive impact of increasing consumer demand for niche brand products b and reducing the cost of live-streamer efforts is greater than the negative impact of reducing consumer demand for well-known brand products a. In this case, a certain degree of product competition is beneficial to the live-streamer, and the profit of the live-streamer will increase with the increase of θ. When $\theta^{*}<\theta <1$, that is, the competition between products is relatively fierce, the negative impact caused by the reduction of demand for well-known brand products a is greater than the positive impact caused by the increase of demand for niche brand products b and the reduction of live-streamer effort cost. At this time, fierce product competition will be detrimental to the live-streamer, and the live-streamer’s profit will decrease with the increase of θ. Therefore, when the live-streamer adopts the two live show strategies of product information disclosure, attention should be paid to the brand differences between the two products. It is not appropriate to choose two products with large brand differences or two products with small brand differences.

Lemma 2 (iii) shows that when the live-streamer puts the product on two live shows but does not disclose the product information at the same time, the profit of the live-streamer will increase with the increase of θ. In the two live shows without product information disclosure (model DN-FS), the demand, price and live-streamer effort level of the well-known brand product a in the first live show are independent of θ. For the niche brand product b in the second live show, as θ increases, the utility obtained by consumers from the niche brand product b increases, and the demand for the niche brand product b will also increase. At the same time, the effort cost of the live-streamer will decrease with the increase of θ. Therefore, in the two live shows (model DN-FS) where product information is not disclosed, the competition between products is beneficial to the live-streamer, and the profit of the live-streamer will increase with the increase of θ. Intuitively speaking, when the brand difference between the two products is smaller, the competition between the products will be more intense, which is not beneficial for the live-streamer who needs to sell the two products. However, in the two live shows (model DN-FS) where product information is not disclosed, when the live-streamer puts competitive similar products with brand differences on two live shows but discloses product information at different times, the fierce competition between products is more beneficial to the live-streamer.

**Lemma 3.** $\pi_{L}^{S}$ and $\pi_{L}^{DC-FS}$ increase with the increase of s. When $s=s^{*}$, $\pi_{L}^{DN-FS}$ is the largest.

Lemma 3 shows that in the two live shows (model S, model DC-FS) under single live show and product information disclosure, the higher the waiting cost of non-fan consumers, that is, the longer the interval between the two live shows, the higher the profit obtained by the live-streamer. Compared with the two live shows with product information disclosure (Model DC-FS) and the two live shows without product information disclosure (Model DN-FS), in the single live show (Model S), when the live-streamer sells the product in a single live show, all consumers can buy the product in the live show without waiting for the next live show. This will lead to savings in the waiting cost of non-fan consumers, and the product demand of non-fan consumers in a single live show will increase. With the increase of s, the longer the interval between the two live shows, the greater the benefit of waiting cost saving under the single live show strategy. Therefore, the profit of the live-streamer will increase with the increase of s.

In the two live shows of product information disclosure (model DC-FS), the live-streamer puts the product in two live shows and discloses the product information at the same time. In this case, the profit of the live-streamer will increase with the increase of s. In the two live shows of product information disclosure, the longer the live show interval, the higher the waiting cost, and the greater the benefit of non-fan consumers from waiting for cost savings in the first live show. Therefore, the more non-fan consumers choose to buy well-known brand products a in the first live show, that is, the demand for well-known brand products a in the first live show increases with the increase of s. However, non-fan consumers who choose to buy niche brand products b in the second live show will decrease, that is, the demand for niche brand products b in the second live show decreases with the increase of s. The positive impact of the increase in consumer demand for well-known brand products a in the first live show is greater than the negative impact of the decrease in consumer demand for niche brand products b in the second live show. Therefore, in the two live shows of product information disclosure (model DC-FS), the profit of the live-streamer will increase with the increase of s.

In the two live shows (model DN-FS) where the product information is not disclosed, the live-streamer puts the product in two live shows but discloses the product information at different times. In this case, when $0<s<s^{*}$, that is, the interval between the two live shows is short, the profit of the live-streamer will increase with the increase of s; when $s>s^{*}$, that is, the interval between the two live shows is long, the profit of the live-streamer will decrease with the increase of s. In the two live shows where product information is not disclosed, as s increases, the higher the waiting cost, the greater the benefits of non-fan consumers due to waiting for cost savings in the first live show. Non-fan consumers’ demand for well-known brand products a in the first live show will increase, while the demand for minority brand products b in the second live show will decrease. When the interval between live shows is short, the positive impact of the increase in demand for well-known brand products a in the first live show is greater than the negative impact of the decrease in demand for minority brand products b in the second live show. At this time, the profit of the live-streamer will increase with the increase of s; In reality, live-streamers often set an optimal live show interval for themselves according to their own conditions. For example, the live show interval of live-streamers such as Li Jiaqi is one day, while the live show interval set by some live-streamers is two days, three days or even longer.

**Lemma 4.** $\pi_{L}^{DC-FS}$,$\pi_{L}^{DN-FS}$ increase with the increase of ρ.

In the two live shows with product information disclosure and the two live shows without product information disclosure (model DC-FS, model DN-FS), the profit of the live-streamer will increase with the increase of ρ, that is, the increase of the live-streamer’s ability to transfer powder. This conclusion is more intuitive. In the process of watching the live show, non-fan consumers will have a certain probability to become fans of the live-streamer because they like the live-streamer itself and its live style, or because the products sold by the live-streamer live show are inexpensive. The stronger the live-streamer’s ability to convert fans, the more the number of non-fan consumers transforming into fan consumers, and the more the number of fan consumers in the live-streamer live room. Due to the different purchase behaviors of fan consumers and non-fan consumers in live show, compared with non-fan consumers, fan consumers are more likely to purchase products in live show rooms and will generate more demand. Therefore, the stronger the ability to convert the powder, the more the live-streamer’s fan-type consumers, the higher the product sales in the live show room, and the higher the profit obtained by the live-streamer in the live show.

## 5. The live-streamer’s strategy selection

### 5.1 Strategy selection of live show

By comparing the profits of the live-streamer in the single live show (Model S) and the two live shows with product information disclosure (Model DC-FS), the single live show (Model S) and the two live shows without product information disclosure (Model DN-FS), the strategy selection of the live-streamer’s live show is studied. In order to reflect the real situation more comprehensively, this paper sets more parameters in the model. In order to analyze the optimal strategy selection of the live-streamer in different situations, this paper sets the parameters that do not consider the impact on the live-streamer in the model to a fixed value. In reality, the commission ratio of the live-streamer in the live show is generally 20%, and the live show fans conversion rate after each live show is generally 5%. For example, Dong Yuhui, the China well-known e-commerce live-streamer, grasp around 2.75 million fans over around 54.31 million views, resulting a 5% fans conversion rate [25]. Therefore, for tractability, we assume that γ=0.2, ρ=0.05, F=1, s=0.4, a=1.2, and the effort cost coefficient of the live-streamer is k=0.5.

Lemma 5. (i) When $\theta <\theta_{a}(\phi )$, $e^{S*}>(e_{a}^{DC-FS*}+e_{b}^{DC-FS*}\backslash )$, and if $\theta \geq \theta_{a}(\phi )$, $e^{S*}\leq \left(e_{a}^{DC-FS*}+e_{b}^{DC-FS*}\right)$; (ii) When $\theta <\theta_{b}(\phi ),e^{S*}>(e_{a}^{DN-FS*}+e_{b}^{DN-FS*},andif\theta \geq \theta_{b}(\phi ),e^{S*}\leq \left(e_{a}^{DN-FS*}+e_{b}^{DN-FS*}\right);$

**Fig 2:**
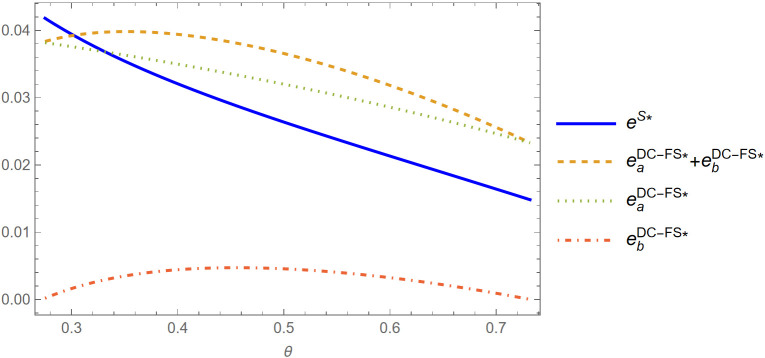
Comparison of parameter *e* between S and DC-FS strategies (*φ* = 0.2) (Prepared by the authors).

**Fig 3:**
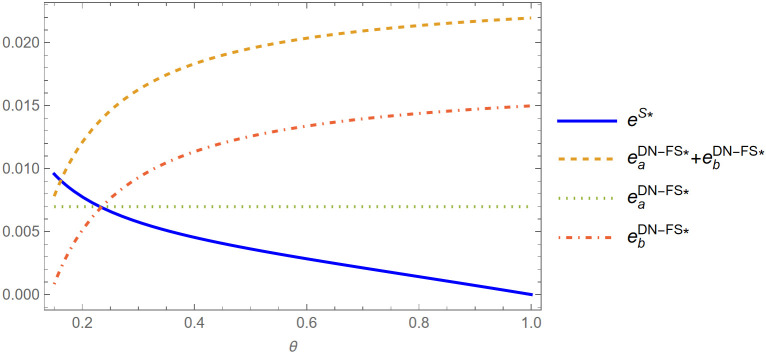
Comparison of parameter *e* between S and DN-FS strategies (*φ* = 0.03) (Prepared by the authors).

Lemma 5 shows that when the brand difference is sufficiently high (low θ), the live-streamer is motivated to exert more entertainment effort to attract consumers under the S strategy than under either the DC-FS or DN-FS strategy. That is, in this scenario, single live show strategy imposes more pressure on the live-streamer in increasing sales when the well-known brand is significantly more valuable than the small brand. Conversely, as the brand difference shrinks, the total entertainment effort in the two live show strategies (DC-FS or DN-FS) becomes larger, and exceeds the entertainment effort under the single live show strategy at certain point. This is because as the two brands become more homogeneous, it is more beneficial for the live-streamer to sell both products all at once since two live show strategy will cause waiting cost for non-fan-type consumers. Interestingly, as shown in Figs 2–3, in the comparison of parameter e between S strategy and two live show with information disclosure (DC-FS), the entertainment effort difference ($e^{S*}$ and $e_{a}^{DC-FS*}+e_{b}^{DC-FS*}$) first enlarges and then slowly shrinks. However, the entertainment effort difference between the S and DN-FS strategies ($e^{S*}$ and $e_{a}^{DN-FS*}+e_{b}^{DN-FS*}$) constantly enlarging. We also find that with information disclosure in the two live show scenarios, the live-streamer puts more entertainment effort in the first live show than that in the second live show, whereas he exerts more effort in the second live show in the case of without disclosing information in most cases. This suggests that concealing the production information in the second live show can encourage the live-streamer to retain entertainment effort in attracting consumers to buy in the second live show.

**Proposition 1** (i) Under the feasible region (with positive demands and profits), when $\theta_{1}(\varphi )<\theta <1$, $\pi_{L}^{DC-FS}>\pi_{L}^{S}$; when $\theta <\theta_{1}(\varphi )$, $\pi_{L}^{S}>\pi_{L}^{DC-FS}$. (ii) when $\theta_{2}(\varphi )<\theta <1$, $\pi_{L}^{DN-FS}>\pi_{L}^{S}$; $\theta <\theta_{2}(\varphi )$, $\pi_{L}^{S}>\pi_{L}^{DN-FS}$.

**Fig 4:**
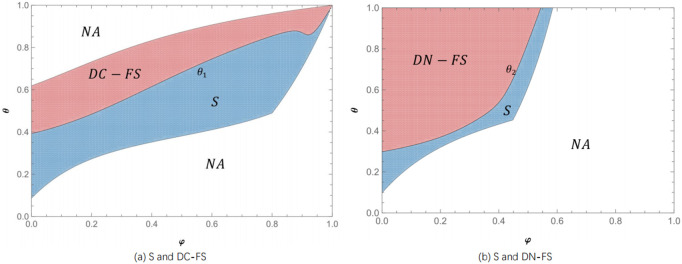
Live-streamer’s live show strategy preference (α = 1.2, *s* = 0.4) (Prepared by the authors).

Proposition 1 shows that when the proportion of fans of the live-streamer is relatively low, regardless of the brand difference between the products, the live-streamer always prefers to sell the products in two live shows. This preference is illustrated in Fig 4, When the proportion of live-streamer fans is relatively small, regardless of the brand difference between products, placing products in two live shows can always weaken the negative impact of competition. Compared with single live show, non-fan consumers will reduce the demand for minority brand products b in the second live show due to the increase of waiting cost in the two live shows. At the same time, in the two live shows, due to the transformation of non-fan consumers, the number of fan consumers in the second live show increased, thus increasing the demand of fan consumers for niche brand products b. In the two live shows, the positive impact of the transformation of non-fan consumers is greater than the negative impact of non-fan consumers due to increased waiting costs. Therefore, when the proportion of fans of the live-streamer is relatively low, the live-streamer prefers to sell the product in two live shows.

When the proportion of fans of the live-streamer is relatively high, regardless of the brand difference between the products, the live-streamer prefers to sell the products in a single live show. As shown in Fig 4, When the number of fans of the live-streamer is large, the number of non-fans of the live-streamer is small. When the live-streamer sells the product in two live shows, the proportion of non-fan-type consumers converted into fan-type consumers will be smaller. The benefits of converting non-fan-type consumers into fan-type consumers in the two live shows will be reduced. The positive impact of non-fan consumers waiting for cost savings in a single live show is greater than the positive impact of non-fan consumers turning into fan consumers in two live shows. Therefore, when the proportion of fans of the live-streamer is relatively high, the live-streamer always likes to sell the product in a single live show.

When the proportion of fans of the live-streamer is medium, with the increase of product competition, the live-streamer is more inclined to choose to sell the product in two live shows. With the reduction of product differences, the competition between products is more intense. The two products with fierce competition are sold in two live shows, which can weaken the negative impact caused by the fierce competition between products. At the same time, the benefits of converting non-fan consumers into fan consumers in the two live shows are greater than the negative effects of non-fan consumers due to increased waiting costs. As illustrated in Fig 4, with the decrease of product differences, live-streamers are more inclined to sell products in two live shows.

### 5.2. The choice of product information disclosure strategy

By comparing and analyzing the profit of the live-streamer under the two live shows of product information disclosure (model DC-FS) and the two live shows of product information undisclosed (model DN-FS), the live-streamer’s information disclosure strategy selection problem is studied. First, we compare the additional utility parameter e under both scenarios.

**Remark 1**. The live-streamer sets a higher entertainment effort in the first live show under the DN-FS strategy than under the DC-FS strategy; The live-streamer sets a higher entertainment effort in the second live show under the DC-FS strategy than that under the DN-FS strategy if θ is relatively low and sets a lower one otherwise. The total entertainment effort set by the live-streamer under the DC-FS strategy is higher than that under the DN-FS strategy when θ is relatively low and vice versa.

**Fig 5:**
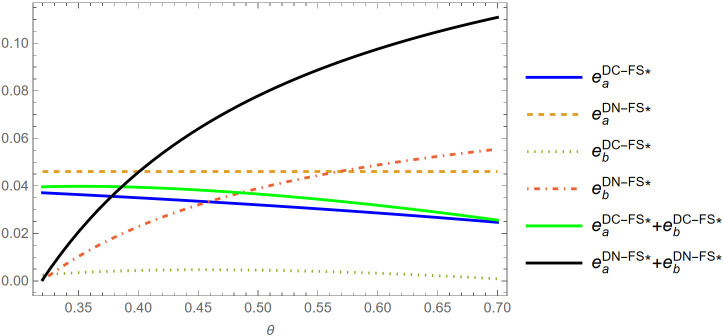
Comparison of parameter *e* between DC-FS and DN-FS strategies (*φ* = 0.2) (Prepared by the authors).

Remark 1 demonstrates the live-streamer’s decision in entertainment effort under two live shows with and without information disclosure. It is worth noting that the live-streamer exerts more entertainment effort in the first live show without information disclosure than that with information disclosure, which is consistent with Lemma 5. Interestingly, as shown in Fig 5, if the brand difference between the two products is sufficiently larger (low θ), the live-streamer exerts more entertainment effort to increase demand under the scenario in which there exists information disclosure of the second live show. Conversely, if the brand difference is small enough, i.e., the brand is sufficiently homogeneous, the live-streamer must devote more entertainment effort under the case of without information disclosing (DN-FS). Furthermore, such entertainment effort difference becomes more significant as the band difference shrinks. This implies that with a significant heterogenous brand products (θ is low), a high capability of entertaining live-streamer is more suitable for the information disclosure strategy.

**Proposition 2** Under the feasible region (with positive demands and profits), when $\theta_{3}(\phi )<\theta <1$, $\pi_{L}^{DN-FS}>\pi_{L}^{DC-FS}$; when $\theta <\theta_{3}(\phi )$,$\pi_{L}^{DC-FS}>\pi_{L}^{DN-FS}$.

Proposition 2 shows that when the live-streamer adopts two live show strategies, when the proportion of fans of the live-streamer is relatively low, the live-streamer prefers to choose the strategy of undisclosed product information. Due to the different purchasing behavior between fan consumers and non-fan consumers in the live show room, fan consumers will purchase all the products in the live show room that make their utility positive, so whether the live-streamer discloses product information has no effect on fan consumers. When the number of fans of the live-streamer is small and the number of non-fans is large, non-fan consumers cannot know the product information of the two live shows at the same time without disclosing product information. Non-fan consumers and fan consumers have the same purchase behavior and will purchase products that make their utility positive. Therefore, the undisclosed product information strategy can weaken the competition between products and increase the demand of non-fan consumers for live show products. Although the disclosure of product information can avoid the demand transfer of non-fan consumers, in the case of a large number of live-streamers and non-fans, the positive impact of weakening product competition under the undisclosed product information strategy is greater than the negative impact caused by demand transfer. Therefore, when the proportion of live-streamer fans is low, it is more beneficial for live-streamers to adopt the strategy of undisclosed product information.

**Fig 6:**
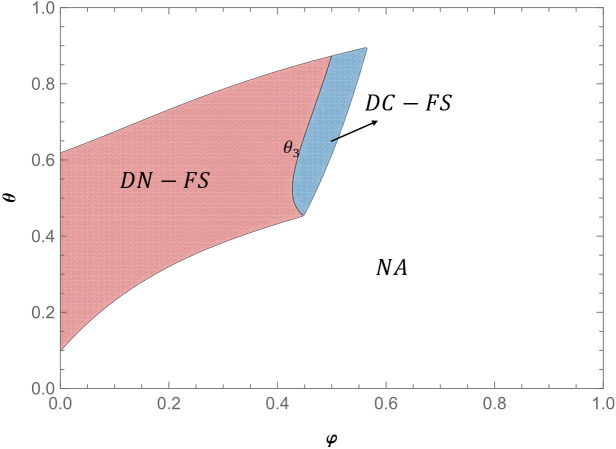
Live-streamer’s product information disclosure strategy preference (*α* = 1.2, *s* = 0.4) (Prepared by the authors).

When the proportion of fans of the live-streamer is relatively high,as depicted in Fig 6. the live-streamer prefers the product information disclosure strategy. When the number of live-streamer fans is large and the number of non-fans is small, the live-streamer’s disclosure of product information can prevent non-fan consumers from transferring their needs in the second live show to the first live show, so that the total demand for products under product information disclosure is higher than the total demand for products without product information disclosure. Although the disclosure of information cannot weaken the negative impact of competition between products, the positive impact of reducing demand transfer under the disclosure strategy is greater than the negative impact of product competition. Therefore, when the proportion of fans is high, it is more beneficial for the live-streamer to adopt the product information disclosure strategy.

When the proportion of fans of the live-streamer is medium, with the increase of product competition, the live-streamer is more inclined to choose the undisclosed product information strategy,as shown in Fig 6.. With the reduction of product differences, the competition between products has become increasingly fierce. The non-disclosure strategy of product information can greatly weaken the negative impact of product competition. At the same time, the positive impact of weakening product competition is greater than the negative impact of demand transfer. Therefore, with the decrease of product differentiation, the live-streamer are more inclined to choose the strategy of undisclosed product information. In reality, live-streamers often adopt the strategy of undisclosed product information, that is, the product information of the next live show is announced after the end of a live show. Even the major live-streamers with relatively large fans such as Li Jiaqi usually adopt the strategy of undisclosed product information. This is because, in reality, the proportion of fans in the live show of live-streamers is often at a medium or relatively low level. Even for major live-streamers, the proportion of fans in the live show cannot reach a relatively high level. At this time, the live-streamer needs to choose whether to disclose product information according to the proportion of fans and the degree of competition between products.

### 5.3 The joint strategy choice of live show and product information disclosure

In reality, live-streamers often face the need to simultaneously decide the number of live shows and information disclosure. Therefore, this section studies the joint strategy selection of live-streamers on live shows and product information disclosure by comparing and analyzing the profits of live-streamers under single live show (Model S), two live shows with product information disclosure (Model DC-FS), and two live shows without product information (Model DN-FS).

**Proposition 3.** Under the feasible region (with positive demands and profits), when $max\{\theta_{3}(\varphi ),\theta_{2}(\varphi \}<\theta <1$, $\pi_{L}^{DN-FS}>{max\{\pi }_{L}^{DC-FS},\pi_{L}^{S}\}$; when $\theta_{1}(\varphi )<\theta <\theta_{3}(\varphi )$, $\pi_{L}^{DC-FS}>{max\{\pi }_{L}^{DN-FS},\pi_{L}^{S}\}$; when $\theta <min\{\theta_{1}(\varphi ),\theta_{2}(\varphi \}$, $\pi_{L}^{S}>{max\{\pi }_{L}^{DC-FS},\pi_{L}^{DN-FS}\}$.

**Fig 7:**
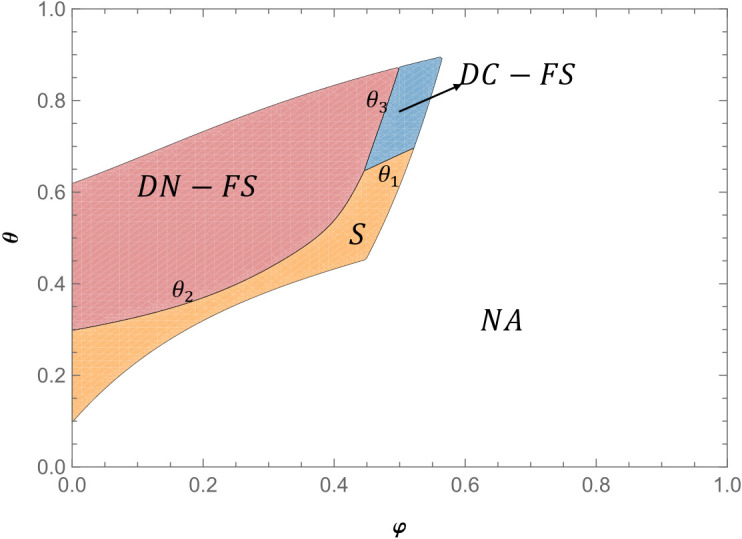
Joint strategy selection considering the proportion of fans and product competition (α = 1.2, *s* = 0.4) (Prepared by the authors).

Proposition 3 shows that when the proportion of live-streamer fans is low, live-streamers prefer to adopt two live show strategies without product information disclosure.,This preference is visually supported by Fig 7. When the number of live-streamer fans is small and the number of non-fans is large, compared with the single live show, although the non-fan consumers will reduce the demand for niche brand productsb in the second live show due to the increase of waiting cost in the two live shows, at the same time, in the two live shows, due to the large number of live-streamer non-fans, a large number of non-fan consumers will be transformed into fan consumers, which will increase the number of fan consumers in the second live show, thus increasing the demand of fan consumers for niche brand products b in the second live show. In the two live shows, the positive impact of the conversion of non-fan consumers is greater than the negative impact of non-fan consumers due to the increase in waiting costs. At the same time, as Proposition 3 shows, the positive impact of weakening product competition under the undisclosed product information strategy is greater than the negative impact caused by demand transfer. Therefore, the live-streamer is more inclined to the two live show strategies under the undisclosed product information. When the proportion of live-streamer fans is medium, with the decrease of product competitiveness, live-streamers are more inclined to the two live show strategies under product information disclosure. When the proportion of fans of the live-streamer is medium, on the one hand, the positive impact of the conversion from non-fan consumers to fan consumers is greater than the negative impact of non-fan consumers due to the increase in waiting costs; on the other hand, the positive impact of reducing demand transfer under the product information disclosure strategy is greater than the negative impact of product competition. Therefore, when the proportion of live-streamer fans is medium, with the decrease of product competitiveness, live-streamers are more inclined to the two live show strategies under product information disclosure.

When the proportion of live-streamer fans is relatively high, with the decrease of product competition, the live-streamer is more inclined to the single live show strategy. As the degree of product competition decreases, the benefits of weakening product competition in the two live shows decrease. At the same time, as Proposition 3 shows, the positive impact of non-fan-type consumers on waiting cost savings in a single live show is greater than the positive impact of non-fan-type consumers converted into fan-type consumers in two live shows. Therefore, when the proportion of fans of the live-streamer is relatively high, the live-streamer always likes to sell the product in a single live show.

**Proposition 4.** When $\theta <\theta_{4}(s)$,$\pi_{L}^{S}>{max\{\pi }_{L}^{DC-FS},\pi_{L}^{DN-FS}\}$; when $\theta_{4}(s)<\theta <\theta_{5}(s)$, $\pi_{L}^{DC-FS}>{max\{\pi }_{L}^{DN-FS},\pi_{L}^{S}\}$; when $\theta_{5}(s)<\theta <1$, $\pi_{L}^{DN-FS}>{max\{\pi }_{L}^{DC-FS},\pi_{L}^{S}\}$.

**Fig 8:**
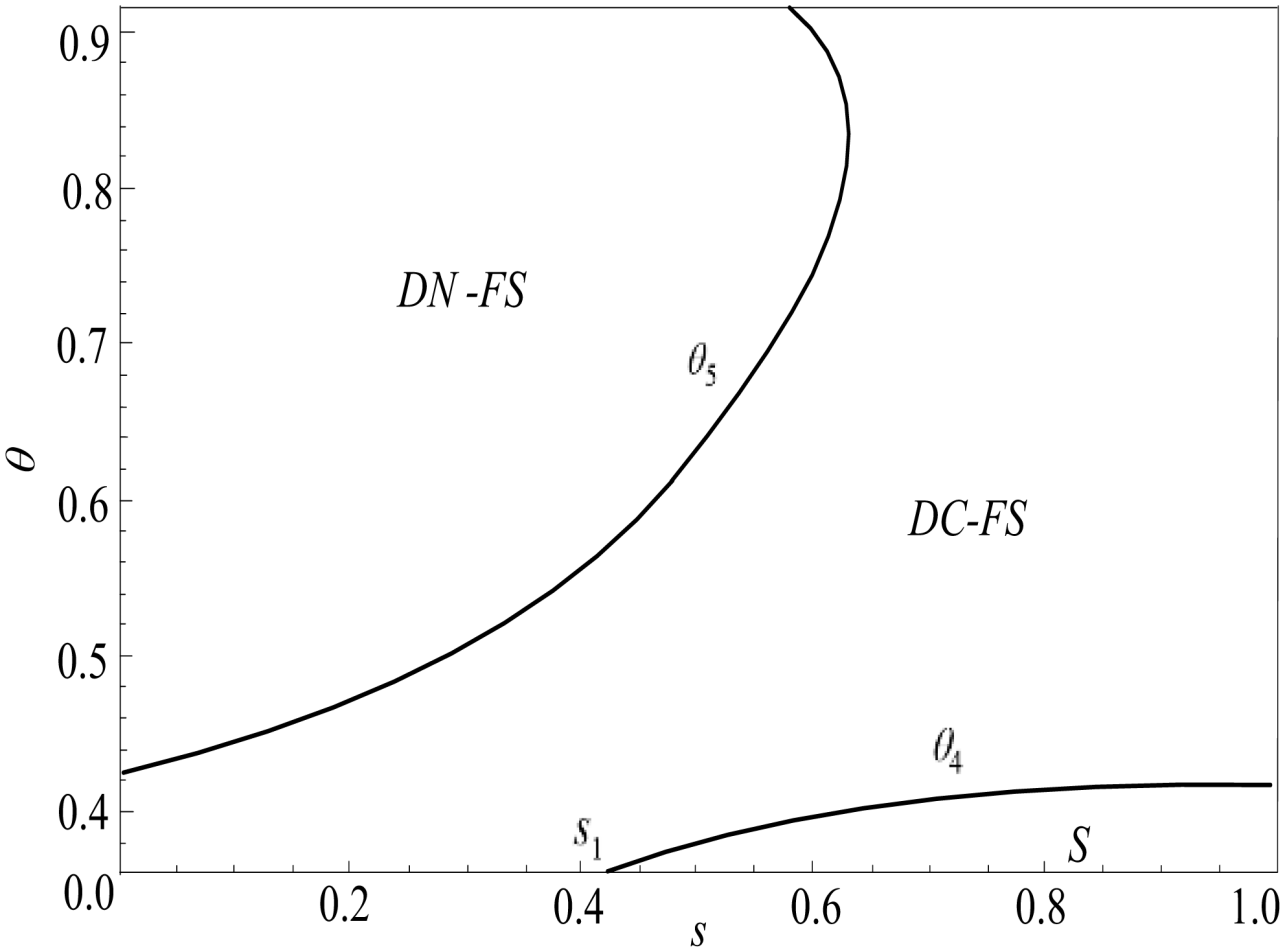
Considering the joint strategy selection of waiting cost and product competition (*α* = 1.2, *φ* = 0.6) (Prepared by the authors).

Proposition 4 shows that the live-streamer prefers to adopt a two-live strategy when the live show interval is short. When the live show interval is short, the benefits of non-fan consumers in a single live show due to not waiting will be very small. At the same time, in the two live shows, some non-fan consumers will be transformed into fan consumers, and the live-streamer will increase the demand for products in the second live show due to the increase in the number of fans in the two live shows. The negative impact of the increase in waiting costs in the two live shows is less than the positive impact of the increase in the number of fans. Therefore, as depicted in Fig 8, when the live show interval is short, the two live show strategy is more beneficial to the live-streamer.

In the case of a short live show interval, with the increase of product competition, the live-streamer are more inclined to adopt two live show strategies with undisclosed product information. With the increase of product competition, non-disclosure of product information can gradually weaken the negative impact of product competition. Although the non-disclosure strategy cannot avoid consumers transferring their needs in the second live show to the first live show, compared with the product information disclosure strategy, the positive impact of weakening product competition under the product information non-disclosure strategy is greater than the negative impact of demand transfer, making the total demand of consumers under the product information non-disclosure strategy greater than the total demand under the disclosure strategy. As illustrated in Fig 8, in the case of a short live show interval, with the increase of product competition, it is more beneficial for the live-streamer to adopt the two live show strategies with undisclosed product information.

In the case of a long live show interval, with the increase of product competition, the live-streamer is also more inclined to adopt two live show strategies with undisclosed product information. With the increase of product competition, the two live show strategies without product information disclosure can weaken the negative impact of product competition to the greatest extent, and this negative impact caused by weakening product competition always dominates. Therefore, in the case of a long live show interval, with the increase of product competition, it is also most beneficial for the live-streamer to adopt the two live show strategies with undisclosed product information.

**Proposition 5.** When $0<\varphi <\phi_{1}(s)$, $\pi_{L}^{DN-FS}>{max\{\pi }_{L}^{DC-FS},\pi_{L}^{S}\}$; when $\phi_{1}(s)<\varphi <\phi_{2}(s)$, $\pi_{L}^{DC-FS}>{max\{\pi }_{L}^{DN-FS},\pi_{L}^{S}\}$; when $\phi_{2}(s)<\varphi <1$, $\pi_{L}^{S}>{max\{\pi }_{L}^{DC-FS},\pi_{L}^{DN-FS}\}$.

**Fig 9:**
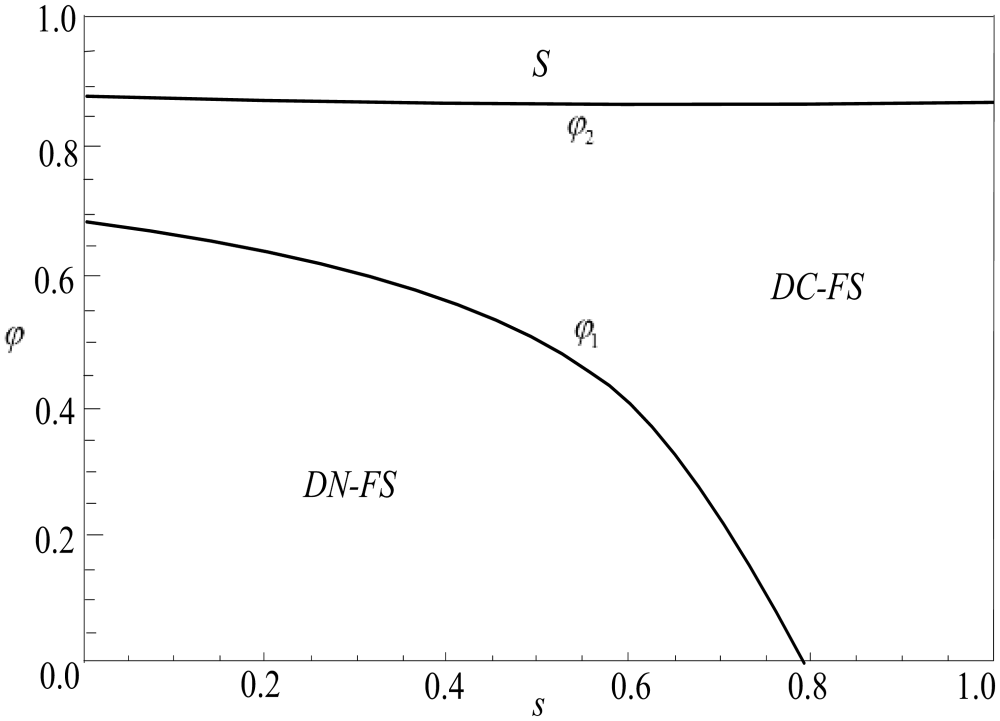
Consider the joint strategy selection of waiting cost and fan ratio (*α* = 1.2, *φ* = 0.6) (Prepared by the authors).

Proposition 5 show that in the case of a low proportion of live-streamer fans, the live-streamer is more inclined to two live show strategies. When the live show interval is short, the live-streamer prefers to adopt the two live show strategies under the undisclosed product information; when the live show interval is long, the live-streamer prefers the two live show strategies under product information disclosure. In the case of a low proportion of live-streamer fans, the positive impact of the transformation of non-fan consumers in the two live shows are always greater than the negative impact of non-fan consumers due to increased waiting costs. Therefore, as depicted in Fig 9, when there are fewer fans, the live-streamer always likes to adopt a two-game live show strategy. When the live show interval is short, the positive impact of weakening product competition under the undisclosed product information strategy is greater than the negative impact of demand transfer. Consumers’ total demand for products without disclosure is always higher than their total demand under the disclosure strategy. At this time, the live-streamer is more inclined to the two live show strategies under the undisclosed product information. When the live show interval is long, the positive impact of avoiding demand transfer under the product information disclosure strategy is greater than the negative impact caused by product competition. At this time, as shown in Fig 9, the live-streamer is more inclined to the two live show strategies under product information disclosure.

In the case of a high proportion of fans, regardless of the length of the interval between the two live shows, the live-streamer prefers a single live show strategy. When the proportion of live-streamer fans is high, the benefits of the live-streamer in the two live shows will be greatly reduced due to the conversion of non-fan consumers into fan consumers. At the same time, in a single live show, non-fan consumers will increase their product demand in a single live show due to waiting cost savings. Therefore, when the proportion of fans of the live-streamer is high, the live-streamer always prefers to adopt a single live show strategy.

## 6. Conclusion

This paper considers the selection of the live-streamer’s live show strategy and product information disclosure strategy when the live-streamer sells two competitive products through live show e-commerce. Based on the reality, this chapter constructs three models: single live show (Model S), two live shows under product information disclosure (Model DC-FS), and two live shows without product information disclosure (Model DN-FS). By solving the optimal decision of the live-streamer under the three models, the profit of the live-streamer under the three different models can be obtained. Through the comparative analysis of the live-streamer under the three models, the main conclusions of this chapter are as follows: (1) When the proportion of fans of the live-streamer is low, the live-streamer prefers the two live show strategies under the undisclosed product information. When the proportion of fans of the live-streamer is high, with the decrease of product competition, the live-streamer is always more inclined to the single live show strategy. When the proportion of live-streamer fans is medium, with the decrease of product competitiveness, the live-streamer is more inclined to the two live show strategies under product information disclosure. (2) In the case of a short live show interval, it is always the most favorable for the live-streamer to adopt a two-field live strategy. With the increase of product competition, live-streamers are more inclined to the two live show strategies without product information disclosure. (3) In the case of a relatively small proportion of live-streamer fans, when the live show interval is short, the live-streamer prefers to adopt the two live show strategies without product information disclosure; when the live show interval is long, the live-streamer prefers the two live show strategies under product information disclosure. In the case of a high proportion of fans, regardless of the length of the interval between the two live shows, the live-streamer prefers a single live show strategy. (4) When the brand difference is sufficiently high, the live-streamer exerts more entertainment effort to attract consumers under the S strategy than under either the DC-FS or DN-FS strategy. If the brand difference between the two products is sufficiently larger, the live-streamer exerts more entertainment effort to increase demand under the DC-FS strategy than under DN-FS strategy.

This paper is established on some strong assumptions and therefore has limitations in deriving further management implications in certain aspects. First, we assume that the number of potential consumers attending the first and second live shows are fixed and equal. However, in practice, the live-streamer may not know the exact number of consumers until the live show begins and thus the potential consumers may be uncertain and inequal between the first and second live shows. Future research can add uncertainty to further explore the live-streamer’s behavior. Second, the commission rate in our paper is assumed to be exogenous and thus we ignore the impact of the live-streamer’s sales revenue-sharing decision, which can be investigated by future studies. Third, the live-streamer may hold more than two live shows to promote products in practice. Future research can extend our study to a multiple live shows situation.

## Supporting information

S1 Appendix (DOCX)

## References

[1] Xiao Y, Yu J, Zhou SX. Commit on Effort or Sales? Value of Commitment in Live-streaming E-commerce. Production and Operations Management. 2024;33(11):2241–58. doi: 10.1177/10591478241270119

[2] Cai J, Wohn DY, Mittal A, Sureshbabu D. Utilitarian and Hedonic Motivations for Live Streaming Shopping. In: Proceedings of the 2018 ACM International Conference on Interactive Experiences for TV and Online Video, 2018. 81–8. doi: 10.1145/3210825.3210837

[3] Wongkitrungrueng A, Assarut N. The role of live streaming in building consumer trust and engagement with social commerce sellers. Journal of Business Research. 2020;117:543–56. doi: 10.1016/j.jbusres.2018.08.032

[4] Su X. An Empirical Study on the Influencing Factors of E-Commerce Live Streaming. In: 2019 International Conference on Economic Management and Model Engineering (ICEMME), 2019. 492–6. doi: 10.1109/icemme49371.2019.00103

[5] Li Y, Li X, Cai J. How attachment affects user stickiness on live streaming platforms: A socio-technical approach perspective. Journal of Retailing and Consumer Services. 2021;60:102478. doi: 10.1016/j.jretconser.2021.102478

[6] Sun Y, Shao X, Li X, Guo Y, Nie K. How live streaming influences purchase intentions in social commerce: An IT affordance perspective. Electronic Commerce Research and Applications. 2019;37:100886. doi: 10.1016/j.elerap.2019.100886

[7] Zhang M, Sun L, Qin F, Wang GA. E-service quality on live streaming platforms: swift guanxi perspective. JSM. 2020;35(3):312–24. doi: 10.1108/jsm-01-2020-0009

[8] Ko H-C, Chen Z-Y. Exploring the Factors Driving Live Streaming Shopping Intention. In: Proceedings of the 7th International Conference on Management of e-Commerce and e-Government, 2020. 36–40. doi: 10.1145/3409891.3409901

[9] Park HJ, Lin LM. The effects of match-ups on the consumer attitudes toward internet celebrities and their live streaming contents in the context of product endorsement. Journal of Retailing and Consumer Services. 2020;52:101934. doi: 10.1016/j.jretconser.2019.101934

[10] Hou J, Shen HC, Xu FS. A model of Livestream Selling with Online Influencers. Available at SSRN 3896924.

[11] Qi AY, Sethi SP, Wei LQ. Top or regular influencer? Contracting in live-streaming platform selling. Available from: https://ssrn.com/abstract=3668390

[12] Baltas G. A model for multiple brand choice. European Journal of Operational Research. 2004;154(1):144–9. doi: 10.1016/s0377-2217(02)00654-9

[13] Luo Z, Chen X, Chen J, Wang X. Optimal pricing policies for differentiated brands under different supply chain power structures. European Journal of Operational Research. 2017;259(2):437–51. doi: 10.1016/j.ejor.2016.10.046

[14] Moorthy S, Chen Y, Tehrani SS. Selling Your Product Through Competitors’ Outlets: Channel Strategy When Consumers Comparison Shop. Marketing Science. 2018;37(1):138–52. doi: 10.1287/mksc.2017.1063

[15] Kuo C-W, Yang S-JS. The role of store brand positioning for appropriating supply chain profit under shelf space allocation. European Journal of Operational Research. 2013;231(1):88–97. doi: 10.1016/j.ejor.2013.05.018

[16] Ru J, Shi R, Zhang J. Does a Store Brand Always Hurt the Manufacturer of a Competing National Brand?. Production and Operations Management. 2015;24(2):272–86. doi: 10.1111/poms.12220

[17] Cui Q, Chiu C-H, Dai X, Li Z. Store brand introduction in a two-echelon logistics system with a risk-averse retailer. Transportation Research Part E: Logistics and Transportation Review. 2016;90:69–89. doi: 10.1016/j.tre.2015.10.005

[18] Ghosh B, Galbreth MR. The Impact of Consumer Attentiveness and Search Costs on Firm Quality Disclosure: A Competitive Analysis. Management Science. 2013;59(11):2604–21. doi: 10.1287/mnsc.2013.1724

[19] Chenavaz RY, Jasimuddin SM. An analytical model of the relationship between product quality and advertising. European Journal of Operational Research. 2017;263(1):295–307. doi: 10.1016/j.ejor.2017.05.016

[20] Guan X, Chen Y-J. Hierarchical quality disclosure in a supply chain with cost heterogeneity. Decision Support Systems. 2015;76:63–75. doi: 10.1016/j.dss.2015.01.002

[21] Johnson JP, Myatt DP. On the Simple Economics of Advertising, Marketing and Product Design. American Economic Review. 2006;93(3):756–84.

[22] Boleslavsky R, Said M. Progressive Screening: Long-Term Contracting with a Privately Known Stochastic Process. The Review of Economic Studies. 2012;80(1):1–34. doi: 10.1093/restud/rds021

[23] Zhang T, Li G, Lai KK, Leung JWK. Information disclosure strategies for the intermediary and competitive sellers. European Journal of Operational Research. 2018;271(3):1156–73. doi: 10.1016/j.ejor.2018.06.037

[24] Li Y, Ning Y, Fan W, Kumar A, Ye F. Channel Choice in Live Streaming Commerce. Production and Operations Management. 2024;33(11):2221–40. doi: 10.1177/10591478241270118

[25] Wu XX. Overnight gain of 3 million followers! Dong Yuhui’s new account premiered with a frenzy of over 100 million yuan, winning over Dongfang Zhenxuan? WangJingShe. 2024 Jan 11 [Cited 2025 February 6]. Available from: https://www.163.com/dy/article/IO5QKMCN0514BOS2.html

