# Supplementary material for: The study on the live streaming frequency strategy choices of streamers in live E-commerce
Source: PLoS One. 2025 Jul 9;20(7):e0324783. doi: 10.1371/journal.pone.0324783 (PMC12240398; doi:10.1371/journal.pone.0324783)
Supplement: S1 Appendix — (DOCX) [file pone.0324783.s001.docx]

**Appendix**

Research on Live Streaming Frequency Strategies and Information Disclosure Strategy Selection for Live Streamers

$A_{1}\left( k,\gamma,\alpha,\theta,\varphi\right)=(2k\alpha^{2}\theta(-1+\varphi)+\gamma(-1+\theta)\varphi^{2})(-2k\alpha^{2}\theta{(-1+\varphi)}^{2}+\gamma(-1+\theta)\varphi^{2})-4(\gamma(-1+\theta)\varphi^{2}+2k\alpha\theta(\alpha{(-1+\varphi)}^{2}+\varphi-\theta\varphi))(\gamma(-1+\theta)\varphi^{2}+2k\alpha(\alpha+\varphi-(\alpha+\theta)\varphi))$,

$A_{2}\left( k,\gamma,\alpha,\theta,\varphi\right)=3\gamma^{2}{(-1+\theta)}^{2}\varphi^{4}+4k^{2}\alpha^{2}\theta(\alpha^{2}(-4+\theta){(-1+\varphi)}^{3}-4\alpha(-1+\theta)(-2+\varphi)(-1+\varphi)\varphi+4{(-1+\theta)}^{2}\varphi^{2})+2k\alpha\gamma(-1+\theta)\varphi^{2}(-4(-1+\theta^{2})\varphi+\alpha(-1+\varphi)(-4-6\theta+5\theta\varphi))$,

$A_{3}\left( k,\gamma,\alpha,\theta,\varphi,\rho\right)=(-1+\gamma)((-1+\rho){(-1+\varphi)}^{2}+\frac{4(\gamma(-1+\theta)\varphi^{2}+k\alpha(\alpha+\varphi-(\alpha+\theta)\varphi))(k\alpha\theta(\alpha+\rho-\alpha\rho-\theta\rho+(-1+\alpha+\theta)(-1+\rho)\varphi)+\gamma(-1+\theta){(\rho+\varphi-\rho\varphi)}^{2})}{k^{2}\alpha^{4}\theta^{2}})$,

$A_{4}\left( k,\gamma,\alpha,\theta,\varphi,\rho\right)=(4\gamma^{2}{(-1+\theta)}^{2}\varphi^{2}{(\rho+\phi-\rho\varphi)}^{2}+k^{2}\alpha^{2}\theta(\alpha^{2}(-4+\theta)(-1+\rho){(-1+\varphi)}^{2}-4\alpha(-1+\theta)(-1+\varphi)(-\rho+2(-1+\rho)\varphi)+4{(-1+\theta)}^{2}\varphi(\rho+\varphi-\rho\varphi))+4k\alpha\gamma(-1+\theta)((-1+\theta)\varphi(-\rho+(-1+\rho)\varphi)(\rho+\varphi+\theta\varphi-\rho\varphi)-\alpha(-1+\varphi)(\rho^{2}-2(-1+\rho)\rho\varphi+(-1+\rho)(-1-\theta+\rho)\varphi^{2})))$,

$A_{5}\left( k,\gamma,\alpha,\theta,\varphi,s,\rho\right)=(-4\gamma^{2}\varphi^{2}{(\rho+\varphi-\rho\varphi)}^{2}+2k^{2}\alpha^{2}\theta(\alpha^{2}(1+s(-1+\varphi)+2\rho(-1+\varphi))(-1+\varphi)+2(\rho(-1+\varphi)-\varphi)\varphi-2\alpha(-1+\varphi)(-\rho+(-1+s+2\rho)\varphi))+4k\alpha\gamma(\varphi(\rho(-1+\varphi)-(1+\theta)\varphi)(-\rho+(-1+\rho)\varphi)-\alpha(-1+\varphi)(\rho^{2}-2(-1+\rho)\rho\varphi+(1-s\theta-(2+\theta)\rho+\rho^{2})\varphi^{2})))$,

$A_{1}^{S}\left( k,\gamma,\alpha,\theta,\varphi,s \right)=2k\alpha(s{(-1+\varphi)}^{2}(\alpha(2+\theta)(-1+\varphi)+2(-1+\theta)\varphi)+\theta(2(-1+\theta)\varphi(1+\varphi)+\alpha(-1+\varphi)(3+(-1+\theta)\varphi)))$,

$A_{1}^{DC-FS}\left( k,\gamma,\alpha,\theta,\varphi,s,\rho\right)=\frac{2(-1+\gamma)(-1+\theta+s(-1+\varphi))(k\alpha\theta(\alpha+\rho-\alpha\rho-\theta\rho+(-1+\alpha+\theta)(-1+\rho)\varphi)+\gamma(-1+\theta){(\rho+\varphi-\rho\varphi)}^{2})}{k\alpha^{2}\theta^{2}}$,

$A_{2}^{DC-FS}\left( k,\gamma,\alpha,\theta,\varphi,s,\rho\right)=(2\gamma(-1+\theta)\varphi^{2}(-s+\rho+s\rho-\theta\rho+(-1-s+\theta)(-1+\rho)\varphi)+k\alpha(2(-1+\theta)\varphi(s-\rho-s\rho+\theta\rho+(1+s-\theta)(-1+\rho)\varphi)+\alpha(-1+\varphi)(s(-1+\rho)(-1+\varphi)+(-1+\theta)(1+\rho+2\varphi-2\rho\varphi))))$,

$A_{1}^{DN-FS}\left( k,\gamma,\alpha,\theta,\varphi,s \right)=(2\gamma\varphi^{2}(s(-1+\varphi)+\varphi)+k\alpha(-2\varphi(s(-1+\varphi)+\varphi)+\alpha(-1+\varphi)(1-s+(2+s)\varphi)))$.

**Proof of the Solution Process for Model S:**

In Model S, Brand 1 and Brand 2 first simultaneously decide their product prices $p_{a}$ and $p_{b}$, and then the live streamer decides their effort level for that particular live show。

Based on formula (4-11) it can be concluded that $\frac{\partial^{2}\pi_{L}^{S}}{\partial e^{2}}=-2k<0$, $\pi_{L}^{S}$is a convex function regarding $e$,  By letting$\frac{\partial\pi_{L}^{S}}{\partial e}=0$, we can obtain the optimal effort level for the streamer$e=\frac{\gamma\varphi(\theta p_{a}+p_{b})}{2k\alpha\theta}$. Substituting$e$into formulas (4-11) and (4-13) respectively, we can derive the results. $\frac{\partial^{2}\pi_{1}^{S}}{\partial{p_{a}}^{2}}=-\frac{\left( 1-\gamma\right)\left( 2k\alpha^{2}\left( 1-\varphi\right)+\left( 2k\alpha-\gamma\varphi\right)\left( 1-\theta\right)\varphi\right)}{k\alpha^{2}\left( 1-\theta\right)}<0，\frac{\partial^{2}\pi_{2}^{S}}{\partial{p_{b}}^{2}}=-\frac{\left( 1-\gamma\right)\left( 2k\alpha^{2}\theta\left( 1-\varphi\right)^{2}+\left( 2k\alpha\theta-\gamma\varphi\right)\left( 1-\theta\right)\varphi\right)}{k\alpha^{2}\left( 1-\theta\right)\theta^{2}}<0$, By letting $\frac{\partial\pi_{1}^{S}}{\partial p_{a}}=0$，$\frac{\partial\pi_{2}^{S}}{\partial p_{b}}=0$, we can find the optimal solutions for $p_{a}^{S}$ and $p_{b}^{S}$. Substituting $p_{a}^{S}$ and $p_{b}^{S}$ into $e$, we can obtain the optimal $e^{S}$。

By substituting the optimal solutions into the demand function and profit function respectively, we can obtain the demand for each product and the streamer's profit under Model S.

$D_{a}^{S}=\left( -\gamma\left( -1+\theta\right)\varphi^{2}+2k\alpha\left( -\alpha+\left( -1+\alpha+\theta\right)\varphi\right) \right)(\gamma(-1+\theta)\varphi^{2}(-2+s{(-1+\varphi)}^{2}+\theta\varphi)+2k\alpha\theta(2(-1+\theta)\varphi+\alpha(-1+\varphi)(2+s{(-1+\varphi)}^{2}+(-2+\theta)\varphi)))/A_{2}\left( k,\gamma,\alpha,\theta,\varphi\right)$,

$D_{b}^{S}=(-\gamma^{2}{(-1+\theta)}^{2}\varphi^{4}(1-2\theta\varphi+s(-2+\varphi+\varphi^{2}))+2k\alpha\gamma(-1+\theta)\varphi^{2}(\theta\varphi(-1+\theta+2\varphi-2\theta^{2}\varphi+\alpha(-1+\varphi)(-5-2\theta+5\theta\varphi))+s(-1+\varphi)(2(-1+\theta^{2})\varphi(1+\varphi)-\alpha(-1+\varphi)(\theta(-2+\varphi)-2(1+\varphi))))+4k^{2}\alpha^{2}\theta(2s(-1+\varphi)(-\alpha+(-1+\alpha+\theta)\varphi)(\alpha{(-1+\varphi)}^{2}-(-1+\theta)\varphi(1+\varphi))+\theta(2{(-1+\theta)}^{2}\varphi^{3}+\alpha^{2}{(-1+\varphi)}^{2}(1+\varphi+(-4+\theta)\varphi^{2})-\alpha(-1+\theta)\varphi(1+\varphi(4+\varphi(-9+4\varphi))))))/\theta A_{2}\left( k,\gamma,\alpha,\theta,\varphi\right)$,

$\pi_{L}^{S}=2F+(k\alpha^{2}\gamma(-1+\theta)(\gamma^{3}{(-1+\theta)}^{3}\varphi^{6}(-11+s^{2}{(-1+\varphi)}^{3}(11+\varphi)+\theta\varphi(14-11\theta\varphi)-2s(-1+\varphi)(7+\varphi(-4-11\theta+5\theta\varphi)))+16k^{3}\alpha^{3}\theta(s^{2}{(-1+\varphi)}^{3}(-\alpha+(-1+\alpha+\theta)\varphi)(\alpha^{2}(4+\theta){(-1+\varphi)}^{3}-4{(-1+\theta)}^{2}\varphi^{2}(1+\varphi)+8\alpha\varphi(-1+\theta+\varphi-\theta\varphi))+2s\theta(-1+\varphi)(-\alpha+(-1+\alpha+\theta)\varphi)(-4{(-1+\theta)}^{2}\varphi^{3}+\alpha^{2}{(-1+\varphi)}^{3}(1+\varphi)(4+(-4+\theta)\varphi)-2\alpha(-1+\theta)(-1+\varphi)\varphi(2+(-1+\varphi)\varphi(-3+2\varphi)))+\theta(4{(-1+\theta)}^{3}\varphi^{3}(1+\theta\varphi^{2})+\alpha^{2}(-1+\theta){(-1+\varphi)}^{2}\varphi(12+\theta+16(-1+\theta)\varphi+(4+(-1+\theta)\theta)\varphi^{2}+2(-6+\theta)\theta\varphi^{3})+\alpha^{3}{(-1+\varphi)}^{3}(4+\theta-8\varphi+6\theta\varphi+(4+\theta(-7+2\theta))\varphi^{2}+(-4+\theta)\theta\varphi^{3})-4\alpha{(-1+\theta)}^{2}(-1+\varphi)\varphi^{2}(-3+\varphi(2+\theta(-2+\varphi)(1+2\varphi)))))+4k\alpha\gamma^{2}{(-1+\theta)}^{2}\varphi^{4}((-1+\theta)\varphi(4+\theta(11-6(1+\theta)\varphi+\theta(11+4\theta)\varphi^{2})-s^{2}{(-1+\varphi)}^{3}(11+3\varphi+4\theta(1+\varphi))-2s(-1+\varphi)(-3+\varphi+\theta(-3+4(3+\theta-\varphi)\varphi)))+\alpha(-1+\varphi)(4+s^{2}{(-1+\varphi)}^{3}(-11-3\theta+(-3+\theta)\varphi)+\theta(10+\varphi(-19+\theta(4+3(7+\theta)\varphi-10\theta\varphi^{2})))-s(-1+\varphi)(2(-3+\varphi)+\theta(4+\varphi(25-16\varphi+2\theta(3+\varphi(-7+5\varphi)))))))-4k^{2}\alpha^{2}\gamma(-1+\theta)\varphi^{2}(s^{2}{(-1+\varphi)}^{3}(-4\alpha(-1+\theta)(-1+\varphi)\varphi(6+8\theta+2\varphi+\theta\varphi)-4{(-1+\theta)}^{2}\varphi^{2}(3+\varphi+4\theta(1+\varphi))+\alpha^{2}{(-1+\varphi)}^{2}(-4(3+\varphi)+\theta(-16-3\theta+3(4+\theta)\varphi)))+2s\theta(-1+\varphi)(\alpha^{2}{(-1+\varphi)}^{2}(-6-11\theta+(-12+(5-3\theta)\theta)\varphi+(10+\theta(22+\theta))\varphi^{2}+2(-9+\theta)\theta\varphi^{3})+4{(-1+\theta)}^{2}\varphi^{2}(1+\varphi(-3-4\theta+\varphi))-2\alpha(-1+\theta)(-1+\varphi)\varphi(1+(12-7\varphi)\varphi+\theta(5+\varphi(13+9(-2+\varphi)\varphi))))+\theta(4{(-1+\theta)}^{2}\varphi^{2}(4+\theta(3+\varphi(-2+3\varphi+4\theta\varphi)))+\alpha^{2}{(-1+\varphi)}^{2}(-16(-1+\varphi)+\theta(15+7\varphi(-2+5\varphi)+\theta\varphi(22+\varphi(-18+3\theta-26\varphi+4\theta\varphi))))-4\alpha(-1+\theta)(-1+\varphi)\varphi(4(-2+\varphi)+\theta(-6+\varphi(6-9\varphi+\theta(-5+\varphi(-8+9\varphi)))))))))/\left( A_{2}\left( k,\gamma,\alpha,\theta,\varphi\right) \right)^{2}$.

To ensure that the research has practical significance, it needs to satisfy the conditions of $p_{a}^{S}>0$, $p_{b}^{S}>0$*,* $e^{S}>0$*,* $D_{a}^{S}>0$*,* $D_{b}^{S}>0$. In reality, the commission rate for streamers is generally 20%. To simplify the model, we let $\gamma=0.2$, and simultaneously set the effort cost coefficient to $k=0.5$. Therefore, in Model S, $\alpha>1$*,* $0.148<\varphi<\frac{1}{6}\left( 1+\sqrt{13} \right)$*,* $\theta_{1}\leq\theta<1$*,* $0<s<1$。

**Proof of the optimal solution of DC-FS：**

In model DC-FS, two brand manufacturers first simultaneously determine product price $p_{a}$ and $p_{b}$, and then the live-streamer decides the effort level $e_{a}$ in the first live show and subsequently, determines the effort level $e_{b}$ in the second live show.

We have $\frac{\partial^{2}\pi_{L2}^{DC-FS}}{\partial e_{b}^{2}}=-k<0$. Letting $\frac{\partial\pi_{L2}^{DC-FS}}{\partial e_{b}}=0$, we have $e_{b}=\frac{\gamma\left( \rho+\varphi-\rho\varphi\right)p_{b}}{2k\alpha\theta}$. Substituting $e_{b}$ into $\pi_{L}^{DC-FS}$, we have$\frac{\partial^{2}\pi_{L}^{DC-FS}}{\partial e_{a}^{2}}=-k<0$. Letting $\frac{\partial\pi_{L}^{DC-FS}}{\partial e_{a}}=0$, we have$e_{a}=\frac{\gamma\varphi p_{a}}{k\alpha}$. We further have $\frac{\partial^{2}\pi_{1}^{DC-FS}}{\partial{p_{a}}^{2}}=-\frac{2\left( 1-\gamma\right)\left( k\alpha^{2}\left( 1-\varphi\right)+\left( k\alpha-\gamma\varphi\right)\left( 1-\theta\right)\varphi\right)}{k\alpha^{2}\left( 1-\theta\right)}<0$. When$\frac{\gamma}{k\alpha}<\theta<1$, $\frac{\partial^{2}\pi_{2}^{DC-FS}}{\partial{p_{b}}^{2}}=-\frac{2\left( 1-\gamma\right)\left( \left( 1-\theta\right)\left( \rho+\varphi-\rho\varphi\right)\left( k\alpha\theta-\gamma\left( \rho+\varphi-\rho\varphi\right) \right)+k\alpha^{2}\theta\left( 1-\rho\right)\left( 1-\theta\right) \right)}{k\alpha^{2}\left( 1-\theta\right)\theta^{2}}<0$. Solving $\frac{\partial\pi_{1}^{DC-FS}}{\partial p_{a}}=0$ and $\frac{\partial\pi_{2}^{DC-FS}}{\partial p_{b}}=0$ yields the optimal$p_{a}^{DC-FS}$ and $p_{b}^{DC-FS}$. Substituting $p_{a}^{DC-FS}$ and $p_{b}^{DC-FS}$ into $e_{a}$ and $e_{b}$, we can obtain the optimal$e_{a}^{DC-FS}$ and $e_{b}^{DC-FS}$.

Substituting the optimal solution into the demand function and profit function, then we can obtain the optimal demand function and profit function under the model DC-FS. Since the expressions of the optimal solution are too complicated, we do not them here.

To ensure our research being meaningful, we limit our attention in the following range: $p_{a}^{DC-FS}>0$, $p_{b}^{DC-FS}>0$, $e_{a}^{DC-FS}>0$, $e_{b}^{DC-FS}>0$, $D_{a}^{DC-FS}>0$, and $D_{b}^{DC-FS}>0$, that is, $\alpha>1$, $0<\varphi<1$, $\frac{0.4}{\alpha}<\theta<1$, and $0<s<1$.

**Proof of the optimal solution of DN-FS：**

In model DN-FS, two brand manufacturers first simultaneously determine product price $p_{a}$ and $p_{b}$, and then the live-streamer decides the effort level $e_{a}$ in the first live show and subsequently, determines the effort level $e_{b}$ in the second live show.

We have $\frac{\partial^{2}\pi_{L2}^{DN-FS}}{\partial e_{b}^{2}}=-k<0$. Letting $\frac{\partial\pi_{L2}^{DN-FS}}{\partial e_{b}}=0$, we have $e_{b}=\frac{\gamma\left( \rho+\varphi-\rho\varphi\right)p_{b}}{k\alpha\theta}$. Substituting $e_{b}$ into $\pi_{L}^{DN-FS}$, we have$\frac{\partial^{2}\pi_{L}^{DN-FS}}{\partial e_{a}^{2}}=-k<0$. Letting $\frac{\partial\pi_{L}^{DN-FS}}{\partial e_{a}}=0$, we have$e_{a}=\frac{\gamma\varphi p_{a}}{k\alpha}$. We further have $\frac{\partial^{2}\pi_{1}^{DN-FS}}{\partial{p_{a}}^{2}}=-\frac{2\left( 1-\gamma\right)\left( k\alpha\left( \alpha+\varphi-\alpha\varphi\right)-\gamma\varphi^{2} \right)}{k\alpha^{2}}<0$. When$\varphi_{1}<\varphi<1$and$\theta_{3}\leq\theta<1$, $\frac{\partial^{2}\pi_{2}^{DN-FS}}{\partial{p_{b}}^{2}}=\frac{\left( 1-\gamma\right)\left( 2k\alpha\theta(s\alpha\left( 1-\varphi\right)+\left( \alpha-1 \right)\rho\left( 1-\varphi\right)-\varphi)+2\gamma\left( \rho+\varphi-\rho\varphi\right)^{2}+\frac{k^{2}\alpha^{4}\theta\left( 1-\varphi\right)\left( 1+s\left( 1-\varphi\right) \right)}{\gamma\varphi^{2}-k\alpha\left( \alpha\left( \alpha-1 \right)\varphi\right)} \right)}{k\alpha^{2}\theta^{2}}<0$. Solving $\frac{\partial\pi_{1}^{DN-FS}}{\partial p_{a}}=0$ and $\frac{\partial\pi_{2}^{DN-FS}}{\partial p_{b}}=0$ yields the optimal$p_{a}^{DN-FS}$ and $p_{b}^{DN-FS}$. Substituting $p_{a}^{DN-FS}$ and $p_{b}^{DN-FS}$ into $e_{a}$ and $e_{b}$, we can obtain the optimal$e_{a}^{DN-FS}$ and $e_{b}^{DN-FS}$.

Substituting the optimal solution into the demand function and profit function, then we can obtain the optimal demand function and profit function under the model DC-FS.

$D_{a}^{DN-FS}=\frac{1}{2}(1+s-s\varphi)$;

$D_{b}^{DN-FS}=\frac{2\gamma\varphi^{2}(s(-1+\varphi)+\varphi)+k\alpha(2\varphi(s-(1+s)\varphi)+\alpha(-1+\varphi)(1-s+(2+s)\varphi))}{4k\alpha(\alpha(-1+\varphi)-\varphi)+4\gamma\varphi^{2}}$;

Since the expressions of the optimal profit are too complicated, we do not them here.

To ensure our research being meaningful, we limit our attention in the following range: $p_{a}^{DN-FS}>0$, $p_{b}^{DN-FS}>0$, $e_{a}^{DN-FS}>0$, $e_{b}^{DN-FS}>0$, $D_{a}^{DN-FS}>0$, and $D_{b}^{DN-FS}>0$, that is, $\alpha>1$, $\varphi_{1}<\varphi<1$, $\theta_{2}\leq\theta<1$, and $0<s<1$.

**Proof of Lemma 1**

In model S, $\frac{\partial e^{S}}{\partial\theta}=(\alpha\gamma\varphi((-1+\theta)(3\gamma^{2}{(-1+\theta)}^{2}\varphi^{4}+4k^{2}\alpha^{2}\theta(\alpha^{2}(-4+\theta){(-1+\varphi)}^{3}-4\alpha(-1+\theta)(-2+\varphi)(-1+\varphi)\varphi+4{(-1+\theta)}^{2}\varphi^{2})+2k\alpha\gamma(-1+\theta)\varphi^{2}(-4(-1+\theta^{2})\varphi+\alpha(-1+\varphi)(-4-6\theta+5\theta\varphi)))(-\gamma\varphi^{2}(1+s{(-1+\varphi)}^{2}-\varphi+2\theta\varphi)+2k\alpha(\alpha(-1+\varphi)(3+s{(-1+\varphi)}^{2}-\varphi+2\theta\varphi)+2\varphi(s{(-1+\varphi)}^{2}+(-1+2\theta)(1+\varphi))))+(3\gamma^{2}{(-1+\theta)}^{2}\varphi^{4}+4k^{2}\alpha^{2}\theta(\alpha^{2}(-4+\theta){(-1+\varphi)}^{3}-4\alpha(-1+\theta)(-2+\varphi)(-1+\varphi)\varphi+4{(-1+\theta)}^{2}\varphi^{2})+2k\alpha\gamma(-1+\theta)\varphi^{2}(-4(-1+\theta^{2})\varphi+\alpha(-1+\varphi)(-4-6\theta+5\theta\varphi)))(-\gamma(-1+\theta)\varphi^{2}(1+s{(-1+\varphi)}^{2}+\theta\varphi)+2k\alpha(s{(-1+\varphi)}^{2}(\alpha(2+\theta)(-1+\varphi)+2(-1+\theta)\varphi)+\theta(2(-1+\theta)\varphi(1+\varphi)+\alpha(-1+\varphi)(3+(-1+\theta)\varphi))))-(-1+\theta)(6\gamma^{2}(-1+\theta)\varphi^{4}+8k^{2}\alpha^{2}(\alpha^{2}(-2+\theta){(-1+\varphi)}^{3}-2\alpha(-1+2\theta)(-2+\varphi)(-1+\varphi)\varphi+2(-1+\theta)(-1+3\theta)\varphi^{2})+2k\alpha\gamma\varphi^{2}(-4(-1+\theta)(1+3\theta)\varphi+\alpha(-1+\varphi)(2-5\varphi+2\theta(-6+5\varphi))))(-\gamma(-1+\theta)\varphi^{2}(1+s{(-1+\varphi)}^{2}+\theta\varphi)+2k\alpha(s{(-1+\varphi)}^{2}(\alpha(2+\theta)(-1+\varphi)+2(-1+\theta)\varphi)+\theta(2(-1+\theta)\varphi(1+\varphi)+\alpha(-1+\varphi)(3+(-1+\theta)\varphi))))))/{(3\gamma^{2}{(-1+\theta)}^{2}\varphi^{4}+4k^{2}\alpha^{2}\theta(\alpha^{2}(-4+\theta){(-1+\varphi)}^{3}-4\alpha(-1+\theta)(-2+\varphi)(-1+\varphi)\varphi+4{(-1+\theta)}^{2}\varphi^{2})+2k\alpha\gamma(-1+\theta)\varphi^{2}(-4(-1+\theta^{2})\varphi+\alpha(-1+\varphi)(-4-6\theta+5\theta\varphi)))}^{2}$. The maximum value of $\frac{\partial e^{S}}{\partial\theta}$ within the feasible region is less than 0. Therefore,$\frac{\partial e^{S}}{\partial\theta}<0$.

In model DC-FS, $\frac{\partial e_{a}^{\mathrm{DC}-FS}}{\partial\theta}=((1-\gamma)\gamma\varphi(-4(k\alpha^{2}\theta^{2}(1-\varphi)(s-\rho-s\rho+\theta\rho+(1+s-\theta)(-1+\rho)\varphi)-2(1+s-\theta-s\varphi)(k\alpha\theta(\alpha+\rho-\alpha\rho-\theta\rho+(-1+\alpha+\theta)(-1+\rho)\varphi)+\gamma(-1+\theta){(\rho+\varphi-\rho\varphi)}^{2}))(2\gamma^{2}(-1+\theta)\varphi^{2}{(\rho+\varphi-\rho\varphi)}^{2}+k^{2}\alpha^{2}\theta(\alpha^{2}(-1+\rho){(-1+\varphi)}^{2}-\alpha(-1+\varphi)(-\rho+2(-1+\rho)\varphi)+(-1+\theta^{2})\varphi(\rho+\varphi-\rho\varphi))+k\alpha\gamma(-(-1+\theta)(\rho(-1+\varphi)-\varphi)\varphi(2\rho(-1+\varphi)-(2+\theta+\theta^{2})\varphi)+\alpha(-1+\varphi)((-2+\theta)\rho^{2}{(-1+\varphi)}^{2}-2\varphi^{2}+\rho\varphi(-4+2\theta+4\varphi-\theta\varphi))))-(-k^{2}\alpha^{4}\theta^{2}(-1+\rho){(-1+\varphi)}^{2}-4(\gamma(-1+\theta)\varphi^{2}+k\alpha(\alpha+\varphi-(\alpha+\theta)\varphi))(k\alpha\theta(\alpha+\rho-\alpha\rho-\theta\rho+(-1+\alpha+\theta)(-1+\rho)\varphi)+\gamma(-1+\theta){(\rho+\varphi-\rho\varphi)}^{2}))(-2\gamma(2-2\theta+s(-2+\theta)(-1+\varphi)){(\rho+\varphi-\rho\varphi)}^{2}+k\alpha\theta(2(-1+\theta^{2}+s(-1+\varphi))(\rho(-1+\varphi)-\varphi)-\alpha(-1+\varphi)(2-2\rho+2s(-1+\rho)(-1+\varphi)+\theta^{2}(\rho+\varphi-\rho\varphi))))))/{(3\gamma^{2}{(-1+\theta)}^{2}\varphi^{4}+4k^{2}\alpha^{2}\theta(\alpha^{2}(-4+\theta){(-1+\varphi)}^{3}-4\alpha(-1+\theta)(-2+\varphi)(-1+\varphi)\varphi+4{(-1+\theta)}^{2}\varphi^{2})+2k\alpha\gamma(-1+\theta)\varphi^{2}(-4(-1+\theta^{2})\varphi+\alpha(-1+\varphi)(-4-6\theta+5\theta\varphi)))}^{2}$. The maximum value of $\frac{\partial e_{a}^{DC-FS}}{\partial\theta}$ within the feasible region is less than 0. Therefore $\frac{\partial e_{a}^{\mathrm{DC}-FS}}{\partial\theta}<0$.

$\frac{\partial e_{b}^{DC-FS}}{\partial\theta}=(\alpha\gamma(\rho+\varphi-\rho\varphi)(\theta(-2\gamma(-2(-1+\theta)(\rho(-1+\varphi)-\varphi)+s(-1+\rho)(-1+\varphi))\varphi^{2}-k\alpha(-2s\varphi+\alpha(-1+\varphi)(-1-\rho+2(-1+\rho)\varphi)+2(2+s-2\theta)\varphi(\rho+\phi-\rho\varphi)))(4\gamma^{2}{(-1+\theta)}^{2}\varphi^{2}{(\rho+\varphi-\rho\varphi)}^{2}+k^{2}\alpha^{2}\theta(\alpha^{2}(-4+\theta)(-1+\rho){(-1+\varphi)}^{2}-4\alpha(-1+\theta)(-1+\varphi)(-\rho+2(-1+\rho)\varphi)+4{(-1+\theta)}^{2}\varphi(\rho+\varphi-\rho\varphi))+4k\alpha\gamma(-1+\theta)((-1+\theta)\varphi(-\rho+(-1+\rho)\varphi)(\rho+\varphi+\theta\varphi-\rho\varphi)-\alpha(-1+\varphi)(\rho^{2}-2(-1+\rho)\rho\varphi+(-1+\rho)(-1-\theta+\rho)\varphi^{2})))-\theta(8\gamma^{2}(-1+\theta)\varphi^{2}{(\rho+\varphi-\rho\varphi)}^{2}+2k^{2}\alpha^{2}(\alpha^{2}(-2+\theta)(-1+\rho){(-1+\varphi)}^{2}-2\alpha(-1+2\theta)(-1+\varphi)(-\rho+2(-1+\rho)\varphi)+2(-1+\theta)(-1+3\theta)\varphi(\rho+\varphi-\rho\varphi))+4k\alpha\gamma((-1+\theta)(\rho(-1+\varphi)-\varphi)\varphi(-2\rho(-1+\varphi)+\varphi+3\theta\varphi)-\alpha(-1+\varphi)(\rho^{2}-2(-1+\rho)\rho\varphi+(-1+\rho)(-2\theta+\rho)\varphi^{2})))(2\gamma(-1+\theta)\varphi^{2}(-s+\rho+s\rho-\theta\rho+(-1-s+\theta)(-1+\rho)\varphi)+k\alpha(2(-1+\theta)\varphi(s-\rho-s\rho+\theta\rho+(1+s-\theta)(-1+\rho)\varphi)+\alpha(-1+\varphi)(s(-1+\rho)(-1+\varphi)+(-1+\theta)(1+\rho+2\varphi-2\rho\varphi))))+(4\gamma^{2}{(-1+\theta)}^{2}\varphi^{2}{(\rho+\varphi-\rho\varphi)}^{2}+k^{2}\alpha^{2}\theta(\alpha^{2}(-4+\theta)(-1+\rho){(-1+\varphi)}^{2}-4\alpha(-1+\theta)(-1+\varphi)(-\rho+2(-1+\rho)\varphi)+4{(-1+\theta)}^{2}\varphi(\rho+\varphi-\rho\varphi))+4k\alpha\gamma(-1+\theta)((-1+\theta)\varphi(-\rho+(-1+\rho)\varphi)(\rho+\varphi+\theta\varphi-\rho\varphi)-\alpha(-1+\varphi)(\rho^{2}-2(-1+\rho)\rho\varphi+(-1+\rho)(-1-\theta+\rho)\varphi^{2})))(2\gamma(-1+\theta)\varphi^{2}(-s+\rho+s\rho-\theta\rho+(-1-s+\theta)(-1+\rho)\varphi)+k\alpha(2(-1+\theta)\varphi(s-\rho-s\rho+\theta\rho+(1+s-\theta)(-1+\rho)\varphi)+\alpha(-1+\varphi)(s(-1+\rho)(-1+\varphi)+(-1+\theta)(1+\rho+2\varphi-2\rho\varphi))))))/{(4\gamma^{2}{(-1+\theta)}^{2}\varphi^{2}{(\rho+\varphi-\rho\varphi)}^{2}+k^{2}\alpha^{2}\theta(\alpha^{2}(-4+\theta)(-1+\rho){(-1+\varphi)}^{2}-4\alpha(-1+\theta)(-1+\varphi)(-\rho+2(-1+\rho)\varphi)+4{(-1+\theta)}^{2}\varphi(\rho+\varphi-\rho\varphi))+4k\alpha\gamma(-1+\theta)((-1+\theta)\varphi(-\rho+(-1+\rho)\varphi)(\rho+\varphi+\theta\varphi-\rho\varphi)-\alpha(-1+\varphi)(\rho^{2}-2(-1+\rho)\rho\varphi+(-1+\rho)(-1-\theta+\rho)\varphi^{2})))}^{2}.$ The maximum value of $\frac{\partial e_{b}^{DC-FS}}{\partial\theta}$ within the feasible region is less than 0. Therefore$\frac{\partial e_{b}^{DC-FS}}{\partial\theta}<0$. In model DN-FS, $\frac{\partial e_{a}^{DN-FS}}{\partial\theta}=0$.

$\frac{\partial e_{b}^{DN-FS}}{\partial\theta}=(\alpha\gamma^{2}{(\rho(-1+\varphi)-\varphi)}^{3}(k\alpha(\alpha(-1+\varphi)-\varphi)+\gamma\varphi^{2})(2\gamma\varphi^{2}(s(-1+\varphi)+\varphi)+k\alpha(2\varphi(s-(1+s)\varphi)+\alpha(-1+\varphi)(1-s+(2+s)\varphi))))/{(-2\gamma^{2}\varphi^{2}{(\rho+\varphi-\rho\varphi)}^{2}+k^{2}\alpha^{2}\theta(\alpha^{2}(1+s(-1+\varphi)+2\rho(-1+\varphi))(-1+\varphi)+2(\rho(-1+\varphi)-\varphi)\varphi-2\alpha(-1+\varphi)(-\rho+(-1+s+2\rho)\varphi))+2k\alpha\gamma(\varphi(\rho(-1+\varphi)-(1+\theta)\varphi)(-\rho+(-1+\rho)\varphi)-\alpha(-1+\varphi)(\rho^{2}-2(-1+\rho)\rho\varphi+(1-s\theta-(2+\theta)\rho+\rho^{2})\varphi^{2})))}^{2}$. The maximum value of $\frac{\partial e_{b}^{DN-FS}}{\partial\theta}$ within the feasible region is less than 0. Therefore$\frac{\partial e_{b}^{DN-FS}}{\partial\theta}<0$.

**Proof of Lemma 2：**

In model S, the $\frac{\partial\pi_{L}^{S}}{\partial\theta}$ expression is too long and thus we do not show it here. We can verify that $\frac{\partial\pi_{L}^{S}}{\partial\theta}<0$ holds in the feasible region in model S. In model DC$-FS$, let $\frac{\partial\pi_{L}^{DC-FS}}{\partial\theta}=0$, we have $\theta=\theta^{*}$, and when$0<\theta<\theta^{*}$, we have $\frac{\partial\pi_{L}^{DC-FS}}{\partial\theta}>0$, otherwise, if $\theta^{*}<\theta<1$, then $\frac{\partial\pi_{L}^{DC-FS}}{\partial\theta}<0$. Thus, at $\theta=\theta^{*}$, $\pi_{L}^{DC-FS}$reaches maximum. In model DN-FS, the $\frac{\partial\pi_{L}^{DN-FS}}{\partial\theta}$ expression is too long and thus we do not show it here. We can verify that $\frac{\partial\pi_{L}^{DN-FS}}{\partial\theta}<0$ holds in the feasible region in model DN-FS.

**Proof of Lemma 3：**

In model S, the $\frac{\partial\pi_{L}^{S}}{\partial s}$ expression is too long and thus we do not show it here. We can verify that $\frac{\partial\pi_{L}^{S}}{\partial s}>0$ holds in the feasible region in model S. In model DC$-FS$, let $\frac{\partial\pi_{L}^{DC-FS}}{\partial s}=0$, we have $s=s^{*}$, and when$0<s<s^{*}$, we have $\frac{\partial\pi_{L}^{DN-FS}}{\partial s}>0$, otherwise, if $s^{*}<s<1$, then $\frac{\partial\pi_{L}^{DN-FS}}{\partial s}<0$. Thus, at $s=s^{*}$, $\pi_{L}^{DN-FS}$ reaches maximum.

**Proof of Lemma 4：**

The expressions $\frac{\partial\pi_{L}^{DN-FS}}{\partial\rho}$ and $\frac{\partial\pi_{L}^{DC-FS}}{\partial\rho}$ are too long and thus we do not show them here. We can verify that $\frac{\partial\pi_{L}^{DN-FS}}{\partial\rho}>0$ and $\frac{\partial\pi_{L}^{DC-FS}}{\partial\rho}>0$ hold in the feasible region.

**Proof of Lemma 5：**

Given that $a=1.2$, $k=0.5$, $\gamma=0.2$, $F=1$, $s=0.4$, $\rho=0.05$. We can derive that when $\theta<\theta_{a}(\varphi)$, $e^{S*}>\left( e_{a}^{DC-FS*}+e_{b}^{DC-FS*} \right)$, and if $\theta\geq\theta_{a}(\varphi)$, $e^{S*}\leq\left( e_{a}^{DC-FS*}+e_{b}^{DC-FS*} \right)$, where $\theta_{a}(\varphi)$ is the root of $e^{S*}=\left( e_{a}^{DC-FS*}+e_{b}^{DC-FS*} \right)$. We can also derive that when $\theta<\theta_{b}(\varphi), e^{S*}>\left( e_{a}^{DN-FS*}+e_{b}^{DN-FS*} \right) , and if \theta\geq\theta_{b}(\varphi), e^{S*}\leq\left( e_{a}^{DN-FS*}+e_{b}^{DN-FS*} \right)$, where $\theta_{b}(\varphi)$ satisfies $e^{S*}==\left( e_{a}^{DN-FS*}+e_{b}^{DN-FS*} \right)$.

**Proof of Proposition 1:**

Solving $\pi_{L}^{DC-FS}>\pi_{L}^{S}$ yields ${\theta>\theta}_{1}\left( \varphi\right)$, where $\theta_{1}\left( \varphi\right)$ satisfies $\pi_{L}^{DC-FS}=\pi_{L}^{S}$. Hence, when $\theta_{1}\left( \varphi\right)<\theta<1$,$\pi_{L}^{DC-FS}>\pi_{L}^{S}$; when $\theta<\theta_{1}\left( \varphi\right)$,$\pi_{L}^{S}>\pi_{L}^{DC-FS}$. Solving $\pi_{L}^{DN-FS}>\pi_{L}^{S}$ yields ${\theta>\theta}_{2}\left( \varphi\right)$, where $\theta_{2}\left( \varphi\right)$ satisfies $\pi_{L}^{DN-FS}=\pi_{L}^{S}$. Therefore, when $\theta_{2}\left( \varphi\right)<\theta<1$,$\pi_{L}^{DN-FS}>\pi_{L}^{S}$; $\theta<\theta_{2}\left( \varphi\right)$,$\pi_{L}^{S}>\pi_{L}^{DN-FS}$.

**Proof of Proposition 2:**

Solving $\pi_{L}^{DC-FS}>\pi_{L}^{DN-FS}$ yields $\theta>\theta_{3}(\varphi)$, where $\theta_{3}(\varphi)$ satisfies $\pi_{L}^{DC-FS}=\pi_{L}^{DN-FS}$. Hence, when $\theta_{3}\left( \varphi\right)<\theta<1$,$\pi_{L}^{DN-FS}>\pi_{L}^{DC-FS}$; when $\theta<\theta_{3}\left( \varphi\right)$,$\pi_{L}^{DC-FS}>\pi_{L}^{DN-FS}$.

**Proof of Proposition 3:**

Combining Proposition 1 and Proposition 2, we can derive the results of Proposition 3.

**Proof of Proposition 4.**

Given $\varphi=0.6$, solving $\pi_{L}^{S}>\pi_{L}^{DC-FS}$ yields $\theta<\theta_{4}$, and solving $\pi_{L}^{DC-FS}>\pi_{L}^{DN-FS}$ yields $\theta<\theta_{5}$, where $\theta_{4}$ and $\theta_{6}$ satisfies $\pi_{L}^{S}=\pi_{L}^{DC-FS}$, and $\pi_{L}^{DC-FS}=\pi_{L}^{DN-FS}$, respectively. In summary, we have that when $\theta<\theta_{4}\left( s \right)$,$\pi_{L}^{S}>{max\{ \pi}_{L}^{DC-FS}, \pi_{L}^{DN-FS}\}$; when $\theta_{4}\left( s \right)<\theta<\theta_{5}\left( s \right)$,$\pi_{L}^{DC-FS}>{max\{ \pi}_{L}^{DN-FS}, \pi_{L}^{S}\}$; when $\theta_{5}\left( s \right)<\theta<1$,$\pi_{L}^{DN-FS}>{max\{ \pi}_{L}^{DC-FS}, \pi_{L}^{S}\}$.

**Proof of Proposition 5.**

Given $\theta=0.6$, solving $\pi_{L}^{S}>\pi_{L}^{DC-FS}$ yields $\varphi>\varphi_{2}$, and solving $\pi_{L}^{DC-FS}>\pi_{L}^{DN-FS}$ yields $\varphi>\varphi_{1}$, where $\varphi_{2}$, and $\varphi_{1}$ satisfies $\pi_{L}^{S}=\pi_{L}^{DC-FS}$ and $\pi_{L}^{DC-FS}=\pi_{L}^{DN-FS}$, respectively. In summary, we have that when $0<\varphi<\varphi_{1}\left( s \right)$,$\pi_{L}^{DN-FS}>{max\{ \pi}_{L}^{DC-FS}, \pi_{L}^{S}\}$; when $\varphi_{1}\left( s \right)<\varphi<\varphi_{2}\left( s \right)$,$\pi_{L}^{DC-FS}>{max\{ \pi}_{L}^{DN-FS}, \pi_{L}^{S}\}$; when$\varphi_{2}\left( s \right)<\varphi<1$,$\pi_{L}^{S}>{max\{ \pi}_{L}^{DC-FS}, \pi_{L}^{DN-FS}\}$.
